# Negative Poisson’s ratio polyethylene matrix and 0.5Ba(Zr_0.2_ Ti_0.8_) O_3_–0.5(Ba_0.7_ Ca_0.3_)TiO_3_ based piezocomposite for sensing and energy harvesting applications

**DOI:** 10.1038/s41598-022-26834-3

**Published:** 2022-12-30

**Authors:** Saptarshi Karmakar, Raj Kiran, Chris Bowen, Rahul Vaish, Vishal Singh Chauhan, Zainab Mufarreh Elqahtani, Samia Ben Ahmed, M. S. Al-Buriahi, Anuruddh Kumar, Tae Hyun Sung

**Affiliations:** 1 School of Engineering, Indian Institute of Technology Mandi, MANDI, Kamand, 175075 Himachal Pradesh India; 2 School of Mechanical and Aerospace Engineering, Nanyang Technological University, 50 Nanyang Avenue, Singapore, 639798 Singapore; 3 Department of Mechanical Engineering, University of Bath, Bath, BA2 7AY UK; 4 Department of Physics, College of Science, Princess Nourah Bint Abdulrahman University, P.O. Box 84428, Riyadh, 11671 Saudi Arabia; 5 Departement of Chemistry, College of Sciences, King Khalid University, P.O. Box 9004, Abha, Saudi Arabia; 6 Department of Physics, Sakarya University, Sakarya, Turkey; 7 Center for Creative Convergence Education, Hanyang University, 222, Wangsimni-Ro, Seongdong-Gu, Seoul, 04763 Korea; 8 Department of Electrical Engineering, Hanyang University, 222, Wangsimni-Ro, Seongdong-Gu, Seoul, 04763 Korea

**Keywords:** Engineering, Materials science

## Abstract

Finite element studies were conducted on 0.5Ba(Zr_0.2_ Ti_0.8_) O_3_–0.5(Ba_0.7_ Ca_0.3_)TiO_3_ (BCZT) piezoelectric particles embedded in polyethylene matrix to create a piezocomposite having a positive and negative Poisson's ratio of −0.32 and 0.2. Polyethylene with a positive Poisson's ratio is referred to as non-auxetic while those with negative Poisson's ratio are referred to as auxetic or inherently auxetic. The effective elastic and piezoelectric properties were calculated at volume fractions of (4%, 8% to 24%) to study their sensing and harvesting performance. This study compared lead-free auxetic 0–3 piezocomposite for sensing and energy harvesting with non-auxetic one. Inherently auxetic piezocomposites have been studied for their elastic and piezoelectric properties and improved mechanical coupling, but their sensing and energy harvesting capabilities and behavior patterns have not been explored in previous literatures. The effect of Poisson's ratio ranging between −0.9 to 0.4 on the sensing and energy harvesting performance of an inherently auxetic lead free piezocomposite composite with BCZT inclusions has also not been studied before, motivating the author to conduct the present study. Auxetic piezocomposite demonstrated an overall improvement in performance in terms of higher sensing voltage and harvested power. The study was repeated at a constant volume fraction of 24% for a range of Poisson's ratio varied between −0.9 to 0.4. Enhanced performance was observed at the extreme negative end of the Poisson's ratio spectrum. This paper demonstrates the potential improvements by exploiting auxetic matrices in future piezocomposite sensors and energy harvesters.

## Introduction

The trends in the electronic industries have always been to reduce their size and power requirements while increasing their functionality. Such endeavors have resulted in low-power microelectronic devices, which are gaining widespread applications in electronic industries^1^. Such devices can be powered by a variety of electrical energy storage(EES) devices like batteries, supercapacitors, chemical storage devices, fuel cells etc.^2^. Recent developments like porous natural wood and Li-Na based supercapacitors with high energy density (31.88 ${\mathrm{Whkg}}^{-1}$) can led to prolonged use of this microelectronic devices^3^. Several other recent developments in the field of energy storage have also increased the use of these microelectronic devices^4–8^. In addition to the energy storage devices, however, due to their low power requirements, energy harvesting devices, energy harvested from ambient sources can also be used as an auxiliary power source. A range of energy harvesting techniques such as photovoltaic, piezoelectric, pyroelectric, thermoelectric, and electromagnetic have been explored and reported in the literature^1,9^. Piezoelectric energy harvesters are devices that convert ambient vibrational energy into electrical energy, and vice-versa, and usually operate in a low vibrational frequency range of ~ 1–100 Hz^10–13^. A piezoelectric material, which can convert mechanical energy into electrical energy and vice-versa, is specifically used for this purpose^14^. Piezoelectric materials generally have a remnant polarization due to charge separation in the crystal structure. Under the influence of an externally applied load, a potential difference is produced by the direct piezoelectric effect due to a change in polarization of the material. The opposite phenomenon is often referred to as the converse piezoelectric effect, where a strain is developed due to an applied electric field. A range of piezoelectric materials demonstrates this phenomenon in which piezoelectric materials can be used in bulk material form, as piezoelectric ceramics, or in composite form as piezocomposites. Piezoceramics can exhibit superior properties in terms of high piezoelectric coefficients but are high density, rigid, brittle, and generally have high acoustic impedance. Piezocomposites, therefore, are generally preferred for sensing and energy harvesting applications for their flexibility, toughness, and ease of manufacturing^15–17^, where several such piezocomposites have already been reported in the literature^14,16^. Since piezoelectric energy harvester harvests vibrational energy, a host vibrational device is required to which the piezocomposite can be attached. Kim et al.^18^ reported on the different types of vibrating devices such as stacks, shells, cymbals, and cantilevers. In the present study, a cantilever beam-type energy harvester is considered and the piezocomposite material is attached to the cantilever beam energy harvester either in a unimorph or bimorph configuration^16,17,19,20^.

A range of piezoelectric materials has been reported in the literature such as polycrystalline ceramics, single-crystal materials, etc. Priya et al.^13^ investigated different lead-based and lead-free piezoelectric materials. Lead-based piezoelectric materials such as $\mathrm{Pb}\left({\mathrm{Mg}}_{1/3}{\mathrm{Nb}}_{2/3}\right){\mathrm{O}}_{3}-{\mathrm{PbTiO}}_{3}$ (PMN-PT), $\mathrm{Pb}\left[{\mathrm{Zr}}_{\mathrm{x}}{\mathrm{Ti}}_{1-\mathrm{x}}\right]{\mathrm{O}}_{3}$ (PZT-5A), and $\mathrm{Pb}\left({\mathrm{Zn}}_{1/3}{\mathrm{Nb}}_{2/3}\right){\mathrm{O}}_{3}-\left(6-7\mathrm{\%}\right){\mathrm{PbTiO}}_{3}$ (PZN-PT) are more popular due to their high piezoelectric coefficient. However, lead is toxic both to humans and to the environment. This has led to an interest in the development of non-toxic lead-free piezoelectric materials^21–23^. Lead-free piezoelectric materials such as $\left({\mathrm{Bi}}_{0.5}{\mathrm{Na}}_{0.5}\right){\mathrm{TiO}}_{3}$ [BNT], sodium potassium niobate $\left({\mathrm{K}}_{0.5}{\mathrm{Na}}_{0.5}\right){\mathrm{NbO}}_{3}$ [KNN], bismuth potassium titanate $\left({\mathrm{Bi}}_{0.5}{\mathrm{K}}_{0.5}\right){\mathrm{TiO}}_{3}$ [BKT], and barium zirconate titanate–barium calcium titanate $0.5\mathrm{Ba}\left({\mathrm{Zr}}_{0.2}{\mathrm{Ti}}_{0.8}\right){\mathrm{O}}_{3}-0.5\left({\mathrm{Ba}}_{0.7}{\mathrm{Ca}}_{0.3}\right){\mathrm{TiO}}_{3}$ [BCZT]^24^ have been reported in the literature. The large piezoelectric coefficient of BCZT makes it a promising piezoelectric material for sensing and energy harvesting applications^25^.

Various means have been devised to improve the piezoelectric performance and sensing and energy harvesting capabilities of piezoceramics and piezocomposites. For example, Zhang et al.^26^ reduced the dielectric loss and enhanced the performance of the BCZT material by inducing porosity in the BCZT material. Kiran et al.^27^ improved sensing and energy harvesting performance of a KNLNTS and PVDF-based piezocomposite by imparting porosity into it and Karmakar et al.^28^ also improved the sensing and energy harvesting capabilities of a BCZT piezoceramic using porosity. Wang et al.^29^ enhanced the piezoelectric properties of BCZT ceramics by optimizing the calcination and sintering temperature of the manufacturing process and Karmakar et al.^30^ studied the effect of sintering temperature on sensing and energy harvesting performance of BCZT piezoceramic sintered at different temperatures. For the past three decades, metamaterials have received significant attention from the research community. Metamaterials are superior materials that possess counterintuitive and unusual properties in various aspects such as density^31^, heat transport^32,33^, and negative Poisson's ratio (NPR) material^34,35^. Lakes^36^ was the first to report the existence of a negative Poisson's ratio (NPR) foam structure, and Evans et al.^37^ later coined the term *auxetics* to describe NPR materials.

**Figure 1:**
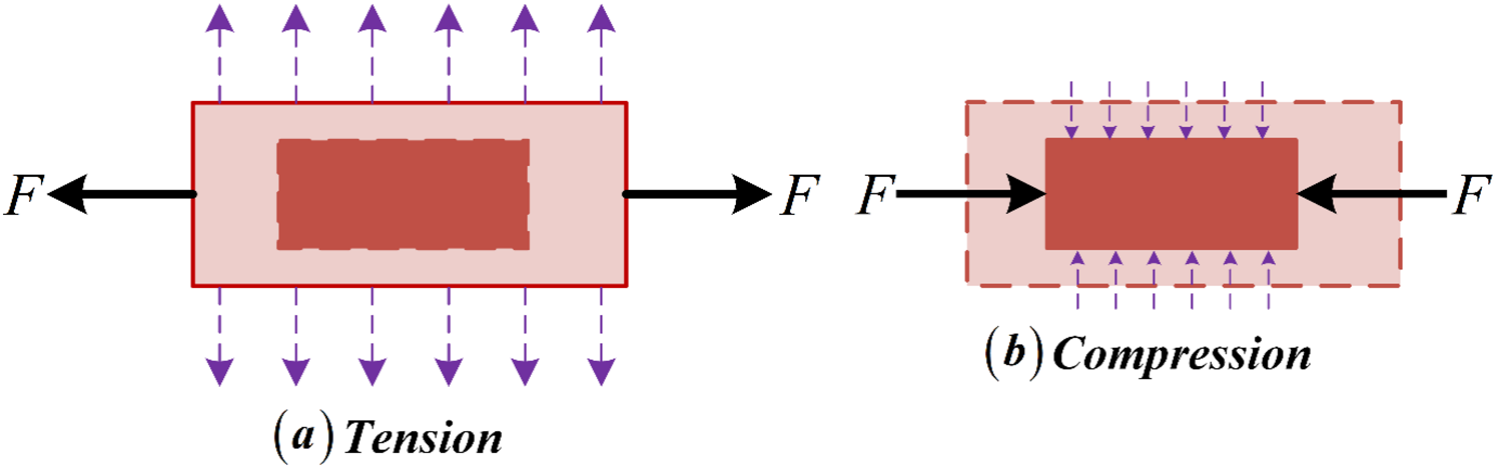
Auxetic material in (**a**) Tension and (**b**) Compression*.*

Auxetic materials are a specific type of material that undergoes expansion when under tension and contraction when under compression, as shown in Fig. 1.

The unusual behavior of auxetic materials imparts many superior properties to the auxetic material such as improved resistance to shear^38^, resistance to indentation^39,40^, resistance to fracture^41^, better energy absorption^42–45^, and better resistance to blasts^46^. Gao et al.^47^ and Li and Shen^48^ demonstrated improved energy absorption capacities of 3 dimensional Voronoi structures and Voronoi foam filled tube using negative Poisson's ratio materials. Lv et al.^49^ reported a novel meta material in which Poisson's ratio can be varied, allowing the materials to be useful for meeting varying requirements for strength and stiffness. Topolov^50^ conducted simulation-based studies on 1–3 piezo-active composites made from an auxetic polymer matrix and found an improvement in the hydrostatic piezoelectric response of the composite. The aforementioned reviews of the literature demonstrate the many ways in which auxetic metamaterials based on negative Poisson's ratio outperform more traditional materials and offer distinct advantages. This has motivated the authors to study the sensing and energy-harvesting potential of piezocomposite materials based on an inherently auxetic polyethylene matrix and containing lead-free BCZT piezoelectric inclusions.

It is possible to impart auxeticity to a material, also known as negative Poisson's ratio behavior, in one of two ways: either by making the material using a novel fabrication route^51–53^, or by using special type of physical structure, such as hierarchical cellular structures, re-entrant unit cell structures, or re-entrant cellular structures, etc., that can impart auxeticity to the material as a whole^54–62^. Krishnaswamy et al.^52^ explored polymeric auxetic matrix-based lead-free 0–3 piezocomposites which demonstrated improved mechanical coupling. They studied the effective homogenized electro-elastic properties of auxetic polyethylene matrix and single-crystal ${\mathrm{BaTiO}}_{3}$ piezoelectric inclusion-based piezocomposite, where an improvement in the effective properties was observed. This has motivated our work to explore the sensing and energy harvesting capabilities of an auxetic polyethylene matrix-based metamaterial using promising lead-free BCZT inclusions to create of lead-free piezocomposite material.

In the present work, a polymer-based inherently auxetic material termed polyethylene (PE)^51^ was used as a matrix with BCZT piezoelectric material as an inclusion to prepare a piezocomposite. Effective properties were calculated considering both positive and negative matrix materials at all the volume fractions.

Various approaches have been reported in the literature to calculate the effecting properties like the analytical approach^63,64^, semi-analytical approaches^65,66^, micro-field approaches^67–69^. Effective properties can also be calculated by applying the homogenization technique to a representative volume element (RVE) and solving by finite element method and has been extensively reported in the literature^70–75^. In the present study, homogenization technique has been used to calculate effective properties. The calculated effective properties of the composite were used further to study the piezocomposite as sensor and energy harvester. The present study explores BCZT based 0–3 piezocomposites with both positive and negative Poisson's ratio matrix-based piezocomposite for this purpose.

## Materials and methods

**Figure 2:**
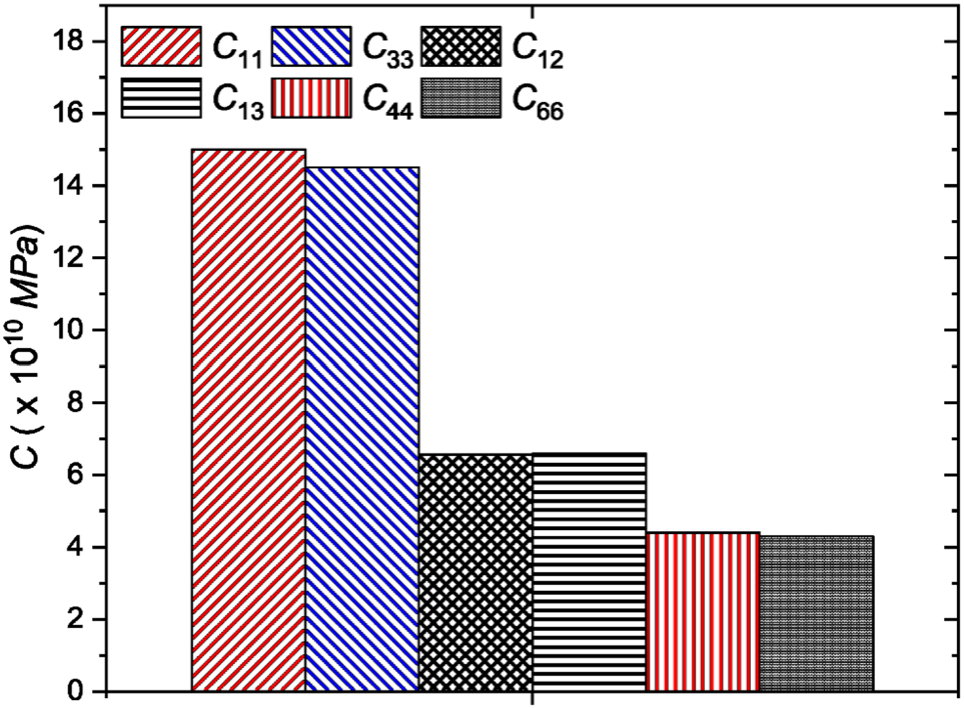
Elastic properties of the BCZT ceramic.

The present work explores the sensing and energy harvesting performance of polyethylene matrix-based piezocomposite with BCZT piezoelectric inclusions. Both negative and positive Poisson's ratio-based polyethylene matrix has been considered in the present study. The polyethylene matrix can be rendered negative or positive Poisson’s ratio depending upon how it has been processed and is often referred to as auxetic and non-auxetic metamaterials. Material properties reported in literature Krishnaswamy et al.^52^ for the matrix material and Zhang et al.^26^ for BCZT piezoelectric inclusions have been used in the present study. The auxetic and non-auxetic matrix made of polyethylene is assumed to have a Poisson's ratio of $-0.32$ and $0.2$ respectively. The elastic modulus is considered to be $E=100\mathrm{ MPa}$^52^. A BCZT piezoelectric material was used as an inclusion to form the 0–3 piezocomposite. The piezoelectric material properties are taken from the literature^26,76,77^ and the elastic material properties are shown in Fig. 2. Piezoelectric coefficients in transverse and longitudinal modes are ${d}_{31}=-298 \mathrm{pC}/\mathrm{N}$ and ${d}_{33}=576 \mathrm{pC}/\mathrm{N}$ and the relative permittivity considered is ${\varepsilon }_{r}=5549$. Six different volume fractions of BCZT inclusion in polyethylene matrix are considered viz. 4%, 8%, 12%, 16%, 20% and 24%. A cantilever beam structure is used to study the sensing and energy harvesting performance of the material. Structural steel was used as a material for the host cantilever beam having Young’s modulus $\mathrm{E}=200 GPa$, and density $\uprho =7850\mathrm{ kg}/{\mathrm{m}}^{3}$.

**Figure 3:**
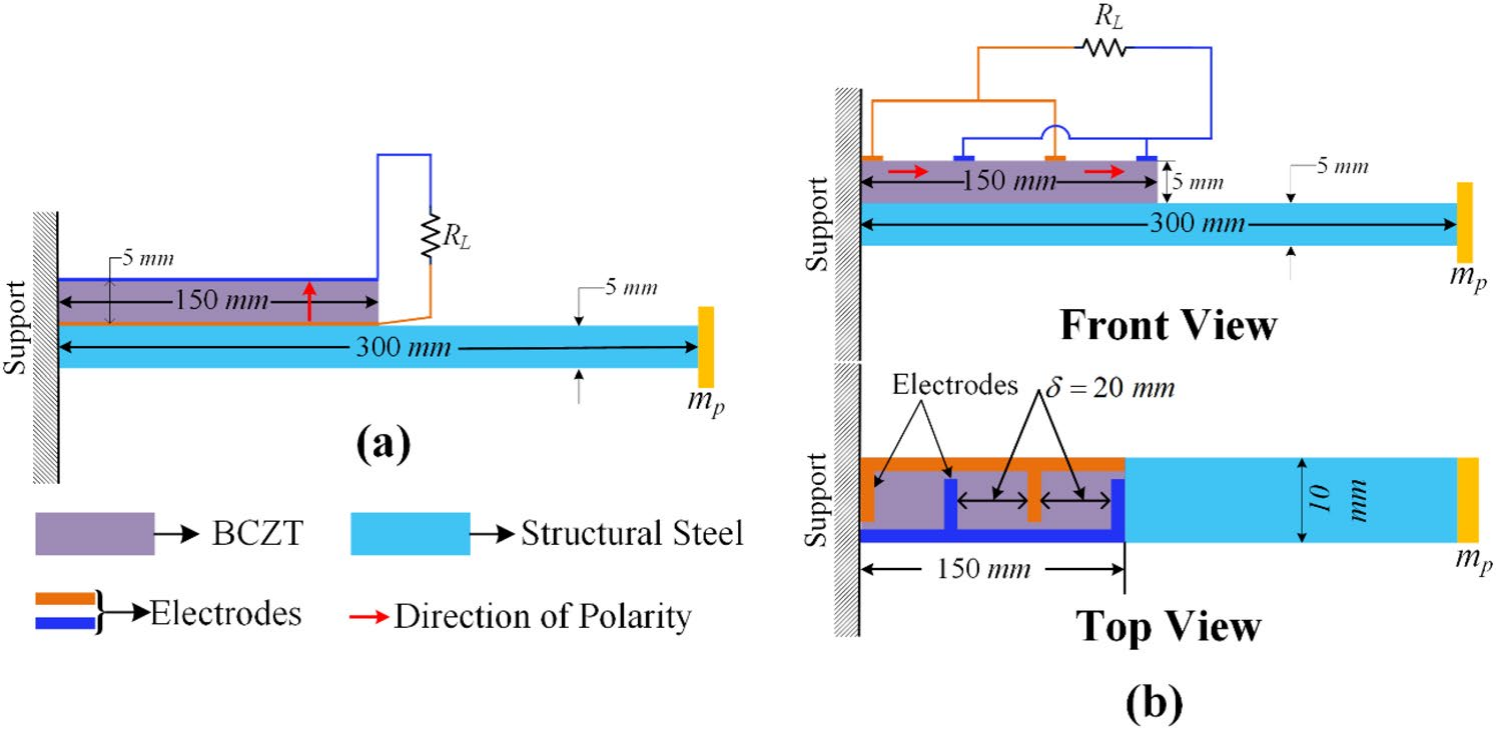
Cantilever beam-based piezoelectric energy harvester operated in (**a**) Transverse mode and (**b**) Longitudinal mode (Front and Top View). “BCZT” in the figure represents 0–3 composite.

An extra proof mass $\left({m}_{p}=15 \mathrm{g}\right)$ is attached to the free end to reduce the natural frequency. Acceleration vibration of $1\times g$ is applied to the base and voltage and power response are collected. The entire structure was subjected to base vibrations in response to which voltage and power are generated and the generated voltage can be used for sensing and the power can be harvested. Fig. 3 shows a schematic diagram of cantilever beam-based piezoelectric energy harvesters, along with their dimensions in millimeters, which are operating in transverse and longitudinal mode.

Energy is harvested by feeding the piezo-current into an external load (a resistance). Generally, as the load resistance varies, the power that can be harvested also varies and maximum power can be obtained at an optimum resistance ${{R}_{L}=R}_{opt}$. The optimum resistance is inversely related to the capacitance between the electrodes [refer to Eq. (1)]

1 $$R_{opt} = \frac{1}{\omega C}$$

where $\omega$ is the first natural frequency and $C$ is the capacitance between the electrodes. Optimum resistance is determined by finding the resistance at which the harvested power becomes maximum.

## Finite element model of the piezocomposite

**Figure 4:**
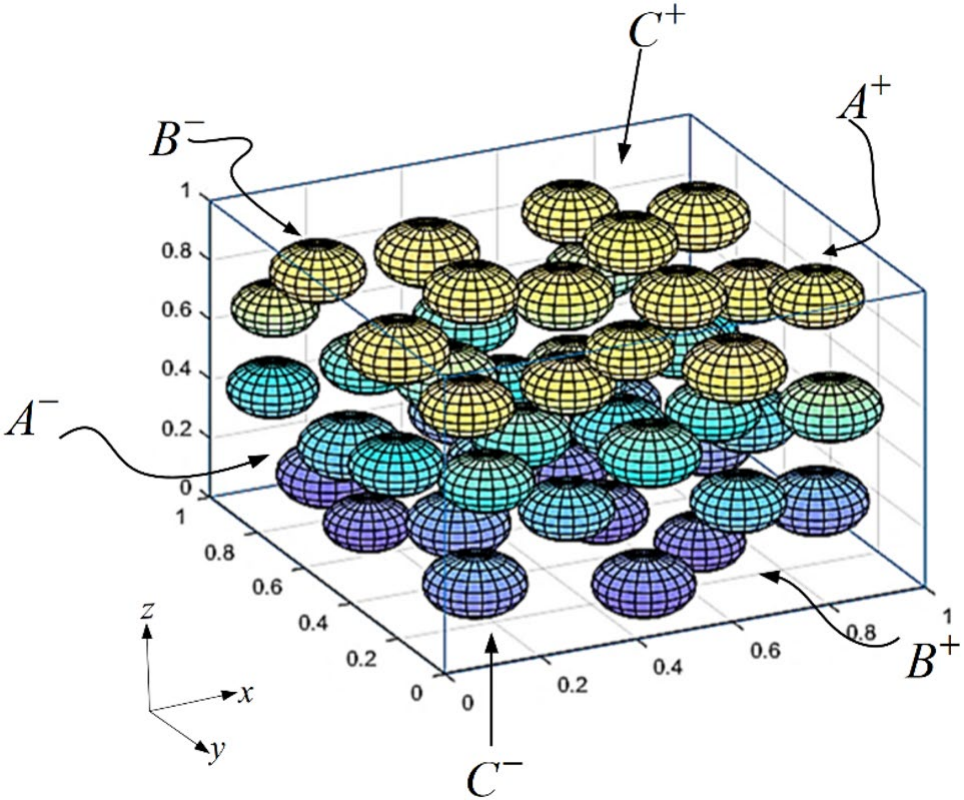
Representative volume element (RVE) containing the piezoelectric inclusions.

The effective elastic and piezoelectric properties of the piezocomposite can be calculated using either the analytical or numerical method^68,78–80^. Analytical methods are suitable for symmetric geometries but are not suitable for arbitrary or complicated shapes. Numerical methods such as the finite element method (FEM) are more suitable to calculate effective properties for piezocomposites having complex shapes and random distribution of inclusions and have been used by several researchers to calculate the effective properties of piezocomposites^80,81^. Berger et al.^72^ evaluated effective properties using both analytical and numerical methods. Following a similar approach, a unit cell model or a representative volume element (RVE) model is used to calculate the effective properties of the composite. RVE is the smallest region or volume of the composite, which represents a homogeneous medium representing the original composite. The process of replacing the original composite with a homogeneous medium is known as homogenization^82^. A typical RVE containing the inclusions within the matrix is shown in Fig. 4. The representative volume element (RVE) used in the current work is thus made up of spherical piezoelectric inclusions that are enclosed within a cubical enclosure made of polyethylene matrix and are embedded within them. The entire assembly, which is a composite, is the RVE that has been used in the current study. In the present study, a MATLAB code has been used to generate the RVE. The random sequential adsorption algorithm, also known as RSA algorithm, is used to position the inclusions inside the RVE. When this algorithm is applied, inclusion particles are introduced randomly within the RVE zone and subsequently adsorbed into the system if they do not interpenetrate with a previously adsorbing inclusion particle. The RSA algorithm is an iterative process which begins with the creation of an inclusion particle within the zone of the unit cell. The iterative process is repeated until the final volume fraction is obtained. The inclusion particle is randomly assigned a position within the unit cell. The next step is to see if the particle's location overlaps with any of the previously placed particles. If there is no overlap, the inclusion particle is absorbed and accepted; otherwise, it is discarded, and the search for a new particle is restarted at random^83–86^. All the particles are added within the unit cell volume of 1 m × 1 m × 1 m to generate the RVE. The size and position data of the inclusion particles within the RVE generated by the MATLAB code are then saved within a MATLAB .mat file. The COMSOL model is saved as a MATLAB file and then the size and position data of the inclusions within the RVE are imported with the help of a *for* loop in MATLAB using MATLAB-COMSOL Live Link. After importing the size and position data, the COMSOL file is generated again which now contains all the inclusions within the RVE. The BCZT inclusions are poled in the $z$-direction and the effective elastic and piezoelectric properties of the composite can be calculated using micromechanical analysis^71^.

Generating a voltage in response to the applied mechanical stress is referred to as the direct piezoelectric effect and the opposite phenomenon is referred to as the converse piezoelectric effect and constitutes a coupled piezoelectric problem. Electromechanical coupling is considered by taking care of both displacement and electrical degree of freedom. The constitutive equation for the coupled piezoelectric problem in stress-charge form is given by Eq. (2).^71^

2 $$\left\{ {\begin{array}{*{20}c} T \\ D \\ \end{array} } \right\} = \left[ {\begin{array}{*{20}c} C & { - e^{t} } \\ e & \varepsilon \\ \end{array} } \right]\left\{ {\begin{array}{*{20}c} S \\ E \\ \end{array} } \right\}$$

The parameters stress $T$ and electrical displacement $D$ to strain $S$ and electric field $E$ are related to each other by material constants $C$ which is the elasticity matrix, $\varepsilon$ the permittivity matrix and $e$ the piezoelectric strain coupling matrix. The term ${e}^{t}$ refers to the transpose of the coupling matrix.

The piezoelectric material is considered to be a transversely isotropic piezoelectric solid, having a hexagonal crystal symmetry. The number of elastic constants reduces from a total of 21 to 5. Equation (2) in its expanded form is given by Eq. (3). Only ten material constants are required to describe the behavior of the material given in the Eq. (3).

3 $$\left\{ {\begin{array}{*{20}c} {\overline{T}_{11} } \\ {\overline{T}_{22} } \\ {\overline{T}_{33} } \\ {\overline{T}_{23} } \\ {\overline{T}_{31} } \\ {\overline{T}_{12} } \\ {\overline{D}_{1} } \\ {\overline{D}_{2} } \\ {\overline{D}_{3} } \\ \end{array} } \right\} = \left[ {\begin{array}{*{20}c} {C_{11}^{eff} } & {C_{12}^{eff} } & {C_{13}^{eff} } & 0 & 0 & 0 & 0 & 0 & { - e_{13}^{eff} } \\ {C_{12}^{eff} } & {C_{11}^{eff} } & {C_{13}^{eff} } & 0 & 0 & 0 & 0 & 0 & { - e_{13}^{eff} } \\ {C_{13}^{eff} } & {C_{13}^{eff} } & {C_{33}^{eff} } & 0 & 0 & 0 & 0 & 0 & { - e_{33}^{eff} } \\ 0 & 0 & 0 & {C_{44}^{eff} } & 0 & 0 & 0 & { - e_{15}^{eff} } & 0 \\ 0 & 0 & 0 & 0 & {C_{44}^{eff} } & 0 & { - e_{15}^{eff} } & 0 & 0 \\ 0 & 0 & 0 & 0 & 0 & {C_{66}^{eff} } & 0 & 0 & 0 \\ 0 & 0 & 0 & 0 & {e_{15}^{eff} } & 0 & {\varepsilon_{11}^{eff} } & 0 & 0 \\ 0 & 0 & 0 & {e_{15}^{eff} } & 0 & 0 & 0 & {\varepsilon_{11}^{eff} } & 0 \\ {e_{13}^{eff} } & {e_{13}^{eff} } & {e_{33}^{eff} } & 0 & 0 & 0 & 0 & 0 & {\varepsilon_{33}^{eff} } \\ \end{array} } \right]\left\{ {\begin{array}{*{20}c} {\overline{S}_{11} } \\ {\overline{S}_{22} } \\ {\overline{S}_{33} } \\ {\overline{S}_{23} } \\ {\overline{S}_{31} } \\ {\overline{S}_{12} } \\ {\overline{E}_{1} } \\ {\overline{E}_{2} } \\ {\overline{E}_{3} } \\ \end{array} } \right\}$$

In the above Eq. (3) $\overline{T }$ is the average value of stress, $\overline{D }$ is the average value of the electric displacement, $\overline{E }$ is the average value of the electric field and $\overline{S }$ is the average value of the strain. $C, e$ and $\varepsilon$ are the elasticity, piezoelectric strain and permittivity. Perfect bonding is assumed between the polyethylene matrix and the spherical BCZT piezoelectric particles used as an inclusion.

### Boundary conditions

Composites can often be represented as a periodic array of RVE’s or unit cells. Therefore, responses are simulated using periodic boundary conditions. The application of a periodic boundary condition ensures that the deformation mode remains the same across the RVE and is devoid of interpenetration and separation between the neighboring RVE. Equation (4) gives the boundary condition in terms of Cartesian coordinates^82,87^.

4 $$u_{i} = \overline{S}_{ij} x_{i} + v_{i}$$

where ${\overline{S} }_{ij}$ is the average engineering strain, ${v}_{i}$ is the global load-dependent local unknown fluctuations. Applying the periodic boundary conditions to the opposite faces of the RVE, the boundary conditions can be written as

5 $$u_{i}^{{K^{ + } }} = \overline{S}_{ij} x_{j}^{K + } + v_{i}^{K + }$$

6 $$u_{i}^{{K^{ - } }} = \overline{S}_{ij} x_{j}^{{K^{ - } }} + v_{i}^{{K^{ - } }}$$

In the above Eqs. (5) and (6) ${K}^{+}$ denotes a direction normal to the $x$ plane in the positive direction and ${K}^{-}$ denotes a direction normal to the $x$ plane in the negative direction denoted by surface ${A}^{-}$ and ${A}^{+}$ in Fig. 4. Similar boundary conditions may be applied to the opposite surfaces ${B}^{-}/{B}^{+}$ and ${C}^{-}/{C}^{+}$.

The local fluctuations on the two opposite faces are identical, therefore, the applied macroscopic strain can be calculated as,

7 $$u_{i}^{{K^{ + } }} - u_{i}^{{K^{ - } }} = \overline{S}_{ij} \left( {x_{j}^{{K^{ + } }} - x_{j}^{{K^{ - } }} } \right)$$

Similarly, for the electric potential, the applied macroscopic electric field is given by,

8 $$\Phi^{{K^{ + } }} - \Phi^{{K^{ - } }} = \overline{E}_{i} \left( {x_{i}^{{K^{ + } }} - x_{i}^{{K^{ - } }} } \right)$$

The stresses, strains, electrical fields, and electrical displacements are volume average over the entire RVE. The corresponding average values are given by,

9 $$\overline{S}_{ij} = \frac{1}{V}\int_{V} {S_{ij} dV}$$

10 $$\overline{T}_{ij} = \frac{1}{V}\int_{V} {T_{ij} dV}$$

11 $$\overline{E}_{i} = \frac{1}{V}\int_{V} {E_{i} dV}$$

12 $$\overline{D}_{i} = \frac{1}{V}\int_{V} {D_{i} dV}$$

In the above Eqs. from (9) to (12), $V$ is the volume of the unit cell. The average properties of the composite are assumed to be the average properties of the unit cell given by Eqs. (9) to (12). The boundary conditions and formulas to calculate different elastic and piezoelectric coefficients are given in Table 1. Boundary conditions are applied such that except one all other field components are zero. The effective properties are calculated by finding the ratio of the average values calculated using Eqs. (9) to (12). Effective properties depend upon the inclusions volume fraction, correspondingly a unit cube can be chosen as an RVE.

**Table 1:** Equations and boundary conditions to calculate effective properties.

Sl. No	Eff. Coeff	$$\begin{gathered} A^{ - } \hfill \\ \left( {u_{i} } \right) \hfill \\ \end{gathered}$$	$$\begin{gathered} A^{ + } \hfill \\ \left( {u_{i} } \right) \hfill \\ \end{gathered}$$	$$\begin{gathered} B^{ - } \hfill \\ \left( {u_{i} } \right) \hfill \\ \end{gathered}$$	$$\begin{gathered} B^{ + } \hfill \\ \left( {u_{i} } \right) \hfill \\ \end{gathered}$$	$$\begin{gathered} C^{ - } \hfill \\ \left( {u_{i} } \right) \hfill \\ \end{gathered}$$	$$\begin{gathered} C^{ + } \hfill \\ \left( {u_{i} } \right) \hfill \\ \end{gathered}$$	Formula
1	${C}_{11}^{eff}$	$0/-$	${u}_{1}/-$	$0/-$	$0/-$	$0/0$	$0$/0	${{\overline{T}_{11} } \mathord{\left/ {\vphantom {{\overline{T}_{11} } {\overline{S}_{11} }}} \right. \kern-0pt} {\overline{S}_{11} }}$
2	${C}_{12}^{eff}$	$0/-$	${u}_{1}/-$	$0/-$	$0/-$	$0/0$	$0/0$	${{\overline{T}_{22} } \mathord{\left/ {\vphantom {{\overline{T}_{22} } {\overline{S}_{11} }}} \right. \kern-0pt} {\overline{S}_{11} }}$
3	${C}_{13}^{eff}$	$0/-$	$0/-$	$0/-$	$0/-$	$0/0$	${u}_{3}/-$	${{\overline{T}_{11} } \mathord{\left/ {\vphantom {{\overline{T}_{11} } {\overline{S}_{33} }}} \right. \kern-0pt} {\overline{S}_{33} }}$
4	${C}_{33}^{eff}$	$0/-$	$0/-$	$0/-$	$0/-$	$0/0$	${u}_{3}$	${{\overline{T}_{33} } \mathord{\left/ {\vphantom {{\overline{T}_{33} } {\overline{S}_{33} }}} \right. \kern-0pt} {\overline{S}_{33} }}$
5	${C}_{44}^{eff}$	${u}_{3}/0$	${u}_{3}/0$	$0/-$	$0/-$	${u}_{1}/-$	${u}_{1}/-$	${{\overline{T}_{13} } \mathord{\left/ {\vphantom {{\overline{T}_{13} } {\overline{S}_{13} }}} \right. \kern-0pt} {\overline{S}_{13} }}$
6	${C}_{66}^{eff}$	${u}_{2}/-$	${u}_{2}/-$	${u}_{1}/-$	${u}_{1}/-$	$0/0$	$0$	${{\overline{T}_{12} } \mathord{\left/ {\vphantom {{\overline{T}_{12} } {\overline{S}_{12} }}} \right. \kern-0pt} {\overline{S}_{12} }}$
7	${e}_{13}^{eff}$	$0/-$	$0/-$	$0/-$	$0/-$	$0/0$	$0/\Phi$	$-{\overline{T} }_{11}/{\overline{E} }_{3}$
8	${e}_{33}^{eff}$	$0/-$	$0/-$	$0/-$	$0/-$	$0/0$	$0/\Phi$	$-{\overline{T} }_{33}/{\overline{E} }_{3}$
9	${e}_{15}^{eff}$	${u}_{3}/0$	${u}_{3}/0$	$0/-$	$0/-$	${u}_{1}/-$	${u}_{1}/-$	${\overline{D} }_{1}/{\overline{S} }_{31}$
10	${\varepsilon }_{11}^{eff}$	$0/0$	$0/\Phi$	$0/-$	$0/-$	$0/-$	$0/-$	${\overline{D} }_{1}/{\overline{E} }_{1}$
11	${\varepsilon }_{33}^{eff}$	$0/-$	$0/-$	$0/-$	$0/-$	$0/0$	$0/\Phi$	${\overline{D} }_{3}/{\overline{E} }_{3}$

### Calculation of Stiffness coefficients

Except for mechanical strain in the first direction, all other strains and electric fields are made zero. Therefore, we have ${\overline{S} }_{11}\ne 0$, but ${\overline{S} }_{22}={\overline{S} }_{33}=\cdots ={\overline{E} }_{2}={\overline{E} }_{3}=0$ [refer to Eq. (3)]. Applying this, ${C}_{11}^{eff}$ and ${C}_{12}^{eff}$ can be calculated using boundary conditions 1 and 2 given in Table 1.

13 $$C_{11}^{eff} = \frac{{\overline{T}_{11} }}{{\overline{S}_{11} }}$$

14 $$C_{12}^{eff} = \frac{{\overline{T}_{22} }}{{\overline{S}_{11} }}$$

Similarly, if ${\overline{S} }_{33}\ne 0$ but ${\overline{S} }_{11}={\overline{S} }_{22}={\overline{S} }_{23}=\cdots ={\overline{E} }_{2}={\overline{E} }_{3}=0$, then ${C}_{13}^{eff}$ and ${C}_{33}^{eff}$ can be calculated using boundary conditions 3 and 4 in Table 1.

15 $$C_{13}^{eff} = \frac{{\overline{T}_{11} }}{{\overline{S}_{33} }}$$

16 $$C_{33}^{eff} = \frac{{\overline{T}_{33} }}{{\overline{S}_{33} }}$$

${C}_{44}^{eff}$ and ${C}_{66}^{eff}$ are calculated by subjecting the RVE to pure shear conditions. Two pairs of opposite surfaces are constrained to give rise to a pure shear condition as given by conditions 5 and 6 given in Table 1. Correspondingly the stiffness coefficients are given by,

17 $$C_{44}^{eff} = \frac{{\overline{T}_{13} }}{{\overline{S}_{31} }}$$

18 $$C_{66}^{eff} = \frac{{\overline{T}_{12} }}{{\overline{S}_{12} }}$$

### Calculation of piezoelectric coefficients and dielectric constants

The effective piezoelectric coefficients ${e}_{13}^{eff}$ and ${e}_{33}^{eff}$ and effective value of the dielectric coefficient ${\varepsilon }_{33}^{eff}$ are calculated by applying zero strains on all the opposing faces and by applying an electrical potential gradient along the $z$ direction. In the present case, a unit voltage is applied on the ${C}^{+}$ surface and a zero strain on all the surfaces of the RVE. The effective coefficients are calculated by applying boundary conditions and formulas given by conditions 7, 8, and 11 in Table 1. Similarly, the dielectric permittivity ${\varepsilon }_{11}^{eff}$ is calculated by applying unit voltage on the surface ${A}^{+}$ and applying zero strains on all other surfaces. Correspondingly, the boundary condition and formula is given by condition 10 in Table 1 is used. The effective property ${e}_{15}^{eff}$ is calculated by applying unit shear strain to opposite faces ${A}^{-}/{A}^{+}$ and ${C}^{-}/{C}^{+}$ as given by condition 9 in Table 1. ${e}_{15}^{eff}$ can be calculated as,

19 $$e_{15}^{eff} = \frac{{\overline{D}_{1} }}{{\overline{S}_{31} }}$$

### Finite element model of the sensor and the energy harvester

The finite element method is often used to undertake static and dynamic analysis of piezoelectric-laminated structures^88–92^. A similar approach has been adopted in the present study to calculate the static and dynamic responses. The finite element domain was discretized using shell elements due to their generalized nature^93–95^. Shell elements, being a generalized element, can take care of curvatures in the finite element domain. First-order shear deformation theory was used to formulate the shell element. Since bending stress is maximum at the fixed end in a cantilever beam configuration, the piezoelectric material is attached closer to the fixed end.

In order to take advantage of the cantilever beam's higher bending stress and bending moment at its fixed end, the piezoelectric material is attached to the cantilever beam beginning at the end of the cantilever beam that is fixed in place. In order to make the analysis more straightforward, a linear form of the piezoelectric theory will be assumed here. An external resistance is connected between the electrodes so that power can be harvested from the system. The electromechanical constitutive equation for piezoelectric material is given by Eq. (2) if the material behaves linearly. The field variables inside the discretized structure can be expressed in terms of the shape function and the nodal variables via the following Eq. (20):

20 $$\left\{ u \right\} = \left[ N \right]\left\{ {q^{e} } \right\}$$

In Eq. (20), the symbols $\left\{u\right\}, \left[N\right]$ and $\left\{{q}^{e}\right\}$ respectively represent the vector of field variables, the shape function, which is also known as the polynomial interpolation function, and the vector of the nodal variables. Because stress is related to strain and strain to displacement, Eq. (21) is often used and can be written in tensorial form given by Eq. (22).

21 $$\left\{ {\varepsilon^{s} } \right\} = \left\{ {\begin{array}{*{20}c} {\frac{\partial u}{{\partial x}}} \\ {\frac{\partial v}{{\partial y}}} \\ {\frac{\partial u}{{\partial y}} + \frac{\partial v}{{\partial x}}} \\ \end{array} } \right\}$$

22 $$\left\{ {\varepsilon^{s} } \right\} = \left[ B \right]_{e} \left\{ {q^{e} } \right\}$$

In Eqs. (21) and (22) $u$ and $v$ are displacements along $x$ and $y$ axis. ${\varepsilon }^{s}$, ${\left[B\right]}_{e}$ and $\left\{{q}^{e}\right\}$ are strain vectors, matrices containing the polynomial function's derivative with respect to material coordinates, and nodal field variable vectors respectively. In a similar fashion, the field variable of the electric field vector has a relationship to the values of the potentials at the nodal coordinates, as shown in the Eq. (23):

23 $$\left\{ E \right\} = \left[ {B_{\phi } } \right]\left\{ \phi \right\}$$

The fundamental equation of motion of an element for the discretized cantilever beam can be derived by applying Hamilton's principle, which is represented by the Eq. (24). This allows for the derivation of the fundamental equation of motion of an element of the cantilever beam.

24 $$\delta \int\limits_{{t_{1} }}^{{t_{2} }} {\left( {K_{E} - H + W^{e} } \right) \times dt} = 0$$

The kinetic energy, potential energy, and electrical energy associated with the piezoelectric material are denoted as ${K}_{E}, H$ and ${W}^{e}$ in Eq. (24). Equation (25) can be used to express kinetic energy and can be expressed as follows

25 $$K_{e} = \frac{1}{2}\int\limits_{V} {\rho \left\{ {\dot{u}} \right\}^{T} \left\{ {\dot{u}} \right\}dV}$$

The expression for kinetic energy can be modified by substituting the expression for nodal displacement, $\left\{u\right\}$, from Eq. (20) into Eq. (25). Equation (26) gives the new expression for kinetic energy and is as follows:

26 $$K^{e} = \frac{1}{2}\left\{ {\dot{q}^{e} } \right\}^{T} \left[ {m_{uu}^{e} } \right]\left\{ {\dot{q}^{e} } \right\}$$

$\left[{m}_{uu}^{e}\right]$ is referred to as the consistent mass matrix in Eq. (26) and is given by Eq. (27).

27 $$\left[ {m^{e} } \right] = \int\limits_{V} {\rho \left[ N \right]^{T} \left[ N \right]dV}$$

Similarly the expression for potential energy term is given by Eq. (28)

28 $$H = \int {\frac{1}{2}\left\{ {\varepsilon^{s} } \right\}^{T} \left\{ \sigma \right\}dV}$$

Piezoelectricity is a coupled phenomenon in which stress $\left\{\sigma \right\}$ is related to the strain $\left\{{\varepsilon }^{s}\right\}$ and the electric field vector $\left\{E\right\}$ using the stiffness coefficient $\left[C\right]$ and piezoelectric coupling coefficient $\left[{e}^{t}\right]$ as given by Eq. (29).

29 $$\left\{ \sigma \right\} = \left\{ {\left[ C \right]\left\{ {\varepsilon^{s} } \right\} - \left[ {e^{t} } \right]\left\{ E \right\}} \right\}$$

In Eq. (29), the mechanical strain and electric field vector can be substituted with values derived from Eqs. (22) and (23). When this particular expression of stress is substituted into Eq. 28, the term for potential energy can be expressed as given by equation

30 $$H = \frac{1}{2}\left\{ {q^{e} } \right\}^{T} \left[ {k_{uu} } \right]\left\{ {q^{e} } \right\} + \frac{1}{2}\left\{ {q^{e} } \right\}^{T} \left[ {k_{u\phi } } \right]\left\{ \phi \right\}$$

The terms $\left[{k}_{uu}\right]$ and $\left[{k}_{u\Phi }\right]$ in Eq. (30) refer to the stiffness terms that are given by Eqs. (31) and (32), and are given below.

31 $$\left[ {k_{uu} } \right] = \int\limits_{V} {\left[ B \right]^{T} \left[ C \right]\left[ B \right]dV}$$

32 $$\left[ {k_{u\phi } } \right] = \int\limits_{V} {\left[ B \right]\left[ e \right]\left[ {B_{\phi } } \right]dV}$$

The electrical energy term is given by Eq. (33)

33 $$W^{e} = \frac{1}{2}\int\limits_{V} {\left\{ E \right\}^{T} \left\{ D \right\}dV}$$

The constitutive equations can be used to rewrite Eq. (33) as given by Eq. (34)

34 $$W^{e} = \frac{1}{2}\left\{ \phi \right\}^{T} \left[ {k_{u\phi } } \right]\left\{ q \right\} + \frac{1}{2}\left\{ \phi \right\}^{T} \left[ {k_{\phi \phi } } \right]\left\{ \phi \right\}$$

Terms $\left[{k}_{u\Phi }\right]$ and $\left[{k}_{\Phi \Phi }\right]$ are the piezoelectric coupling and capacitance matrix. The equation of motion can be obtained by substituting all of the energy terms into Hamilton's equation. Taking into account the material damping, and simplifying the elemental equation, the equation of motion can be written as given by Eqs. (35) and (36)^96^.

35 $$\left[ {M_{uu}^{e} } \right]\left\{ {\ddot{u}^{e} } \right\} + \left[ {C_{uu}^{e} } \right]\left\{ {\dot{u}^{e} } \right\} + \left[ {K_{uu}^{e} } \right]\left\{ {u^{e} } \right\} + \left[ {K_{u\varphi }^{e} } \right]\left\{ {\varphi^{e} } \right\} = \left\{ {f_{ext}^{e} } \right\}$$

36 $$\left[ {K_{\varphi u}^{e} } \right]\left\{ {u^{e} } \right\} + \left[ {K_{\varphi \varphi }^{e} } \right]\left\{ {\varphi^{e} } \right\} = \left\{ {Q^{e} } \right\}$$

In Eqs. (35) ${M}_{uu}^{e}, {C}_{uu}^{e}$ and ${K}_{uu}^{e}$ are the consistent mass, damping and stiffness matrix for an element. ${K}_{u\varphi }^{e}$ and ${K}_{\varphi u}^{e}$ are the coupling matrices for an element. ${K}_{\varphi \varphi }^{e}$ dielectric stiffness matrix for an element. The nodal field variables record the response in terms of displacement $\left\{{u}^{e}\right\}$ and potential $\left\{{\varphi }^{e}\right\}$ at the element. The external force and charges on a single element are given by $\left\{{f}_{ext}^{e}\right\}$ and $\left\{{Q}^{e}\right\}$.

Equation (37) gives the elemental stiffness matrix.

37 $$\left[ K \right]_{e} = \left[ {\begin{array}{*{20}c} {K_{uu}^{e} } & {K_{u\varphi }^{e} } \\ {K_{\varphi u}^{e} } & {K_{\varphi \varphi }^{e} } \\ \end{array} } \right]$$

Global equations, obtained by assembling the elemental equations, are given below in Eqs. (38) and (39).

38 $$\left[ {M_{uu}^{G} } \right]\left\{ {\ddot{u}^{G} } \right\} + \left[ {C_{uu}^{G} } \right]\left\{ {\dot{u}^{G} } \right\} + \left[ {K_{uu}^{G} } \right]\left\{ {u^{G} } \right\} + \left[ {K_{u\varphi }^{G} } \right]\left\{ {\varphi^{G} } \right\} = \left\{ {f_{ext}^{G} } \right\}$$

39 $$\left[ {K_{{_{\varphi u} }}^{G} } \right]\left\{ {u^{G} } \right\} + \left[ {K_{\varphi \varphi }^{G} } \right]\left\{ {\varphi^{G} } \right\} = \left\{ {Q^{G} } \right\}$$

Matrices in Eqs. (38) and (39) are corresponding global matrices obtained after assembly. Equations (38) and (39) are coupled together to achieve the final coupled global equation, as given below in Eq. (40)

40 $$\left[ {\begin{array}{*{20}c} {M_{uu}^{G} } & 0 \\ 0 & 0 \\ \end{array} } \right]\left\{ {\begin{array}{*{20}c} {\ddot{u}^{G} } \\ {\ddot{\varphi }^{G} } \\ \end{array} } \right\} + \left[ {\begin{array}{*{20}c} {C_{uu}^{G} } & 0 \\ 0 & 0 \\ \end{array} } \right]\left\{ {\begin{array}{*{20}c} {\dot{u}^{G} } \\ {\dot{\varphi }^{G} } \\ \end{array} } \right\} + \left[ {\begin{array}{*{20}c} {K_{uu}^{G} } & {K_{u\varphi }^{G} } \\ {K_{\varphi u}^{G} } & {K_{{_{\varphi \varphi } }}^{G} } \\ \end{array} } \right]\left\{ {\begin{array}{*{20}c} {u^{G} } \\ {\varphi^{G} } \\ \end{array} } \right\} = \left\{ {\begin{array}{*{20}c} {f_{ext}^{G} } \\ {Q^{G} } \\ \end{array} } \right\}$$

Equations (35) to (37) represents the equation of motion for an element whereas Eqs. (38) to (40) do the same for the entire discretized domain. The number of unknowns in the elemental Eqs. (35) to (37) is dependent on the number of degrees of freedom defined for each individual element. The number of degrees of freedom is typically defined by the number of nodal field variables that are used to approximate the solution or solutions within an element domain and are constant for all of the elements across the discretized domain. The number of unknowns that need to be solved for in the global Eqs. (38) to (40), on the other hand, is greater than that in the elemental equations, and it is dependent upon the number of elements *N* that were used to discretize the domain. These global equations are produced by assembling the elemental equations. For the convenience of referring to the equations, the superscript $G$ is dropped in all the subsequent equations in the manuscript and is to be understood as global equation.

A Dirichlet boundary condition $\left(u=0\right)$ is applied at the fixed end. Initial values of the displacement and charge distribution are considered to be zero. The system is considered to have zero initial displacements and charge. The system response, measured in terms of open-circuit voltage, is given by Eq. (41). Correspondingly harvested power can be calculated by calculating current and voltage across the resistance using Eqs. (42) to (45).

41 $$\left\{ \varphi \right\} = - \left[ {K_{\varphi \varphi } } \right]^{ - 1} \left[ {K_{\varphi u} } \right]\left\{ u \right\}$$

Current can be calculated from accumulated charge and voltage developed given below in the following equations,

42 $$i = - \frac{dQ}{{dt}}$$

43 $$i = \frac{V}{{R_{L} }}$$

Using the expression of charge from Eqs. (39) and (42), the current is given by,

44 $$i = - \frac{dQ}{{dt}} = - \frac{d}{dt}\left( {\left[ {K_{\varphi u} } \right]\left\{ u \right\} + \left[ {K_{\varphi \varphi } } \right]\left\{ \varphi \right\}} \right)$$

Using Eqs. (43) and (44), the voltage is given by,

45 $$V = R_{L} \times - \frac{d}{dt}\left( {\left[ {K_{\varphi u} } \right]\left\{ u \right\} + \left[ {K_{\varphi \varphi } } \right]\left\{ \varphi \right\}} \right)$$

The power across the load resistance can be calculated using Eqs. (44) and (45) using the relation $P=Vi$. For a much detailed formulation, refer to the authors other paper^97^.

## Results and discussion

**Figure 5:**
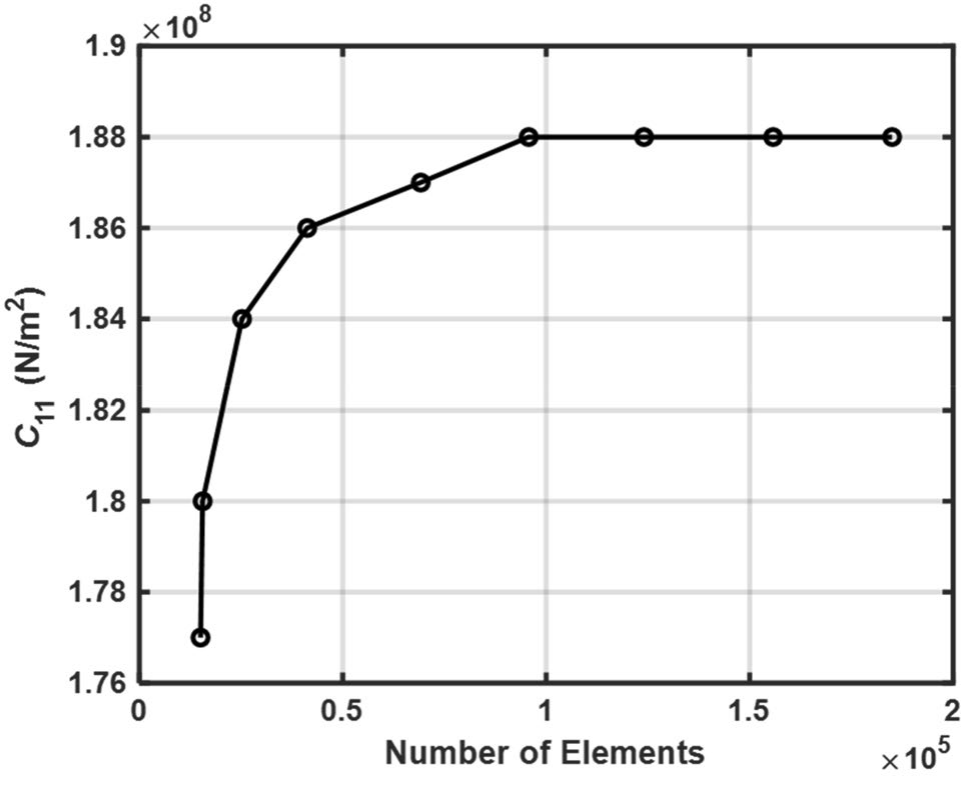
Mesh convergence study to calculate effective property ${C}_{11}$ for non-auxetic piezocomposite at 24% volume fraction.

The homogenization technique is applied to the representative volume element in order to calculate the effective properties of both the auxetic and non-auxetic 0–3 piezocomposite (RVE). The RVE zone is discretized in order to accomplish this goal using a finite number of elements. A gradual increase in the number of elements is performed until there is no longer any discernible shift in the solutions. The coefficients of stiffness of the non-auxetic piezocomposite are shown in the Fig. 5.

These coefficients were calculated at volume fractions of 24% using a variety of elements. After approximately $1.2\times {10}^{5}$ elements have been added, it has been observed that the stiffness coefficient ${C}_{11}$ does not encounter any significant variation. The presence of that many elements helped to discretize the space of the domain in a manner that corresponded to this concept. The same steps were repeated in the calculation of other piezoelectric and elastic effective properties.

As the inclusion volume fraction increased, the effective properties improved which in turn increased the sensing voltage and harvested power. The same study was repeated at 24% volume fraction for a range of Poisson’s ratio $\left(\nu =-0.9 \mathrm{to} 0.4\right)$. Effective properties, sensing voltage, and harvested power all showed significant improvement in the case of the auxetic matrix at the negative extreme of the Poisson's ratio spectrum compared to the positive one.

**Figure 6:**
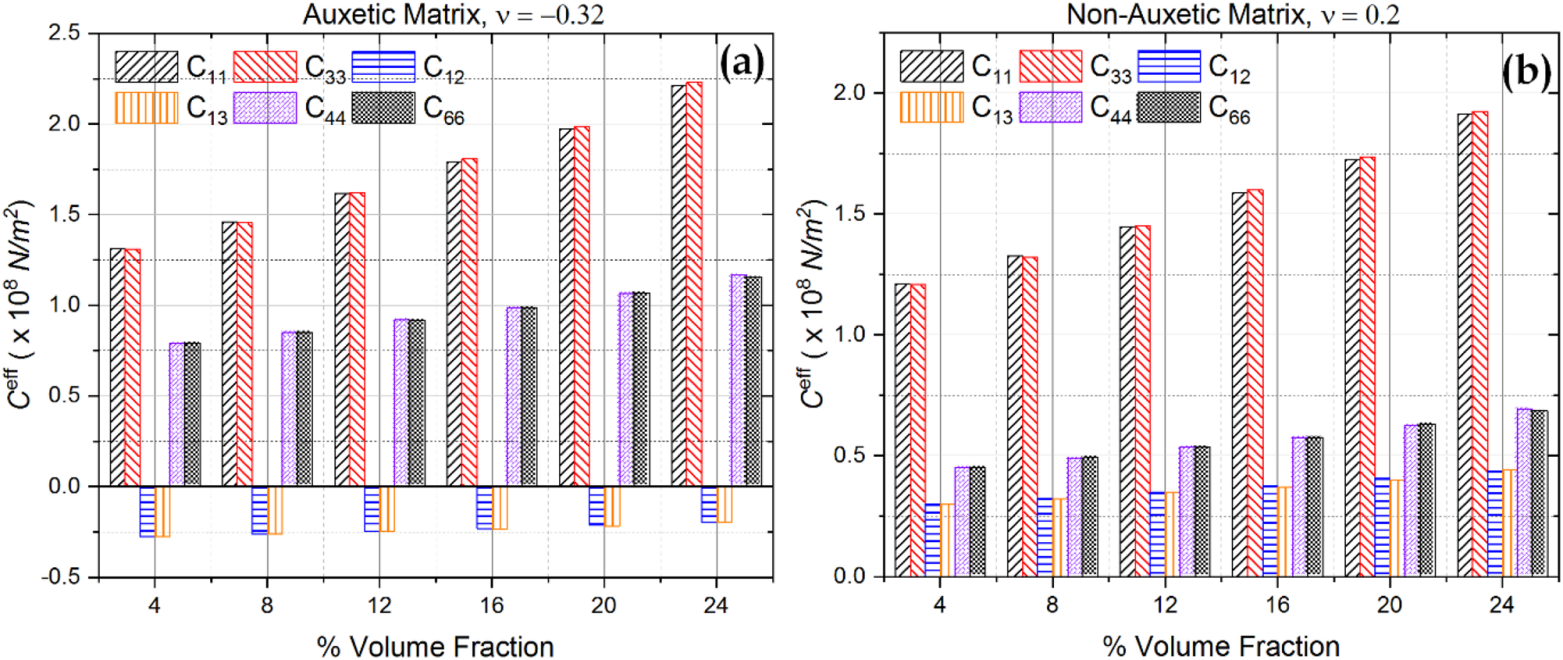
Effective stiffness parameters calculated at different volume fractions (**a**) Auxetic matrix (**b**) non-Auxetic Matrix.

**Figure 7:**
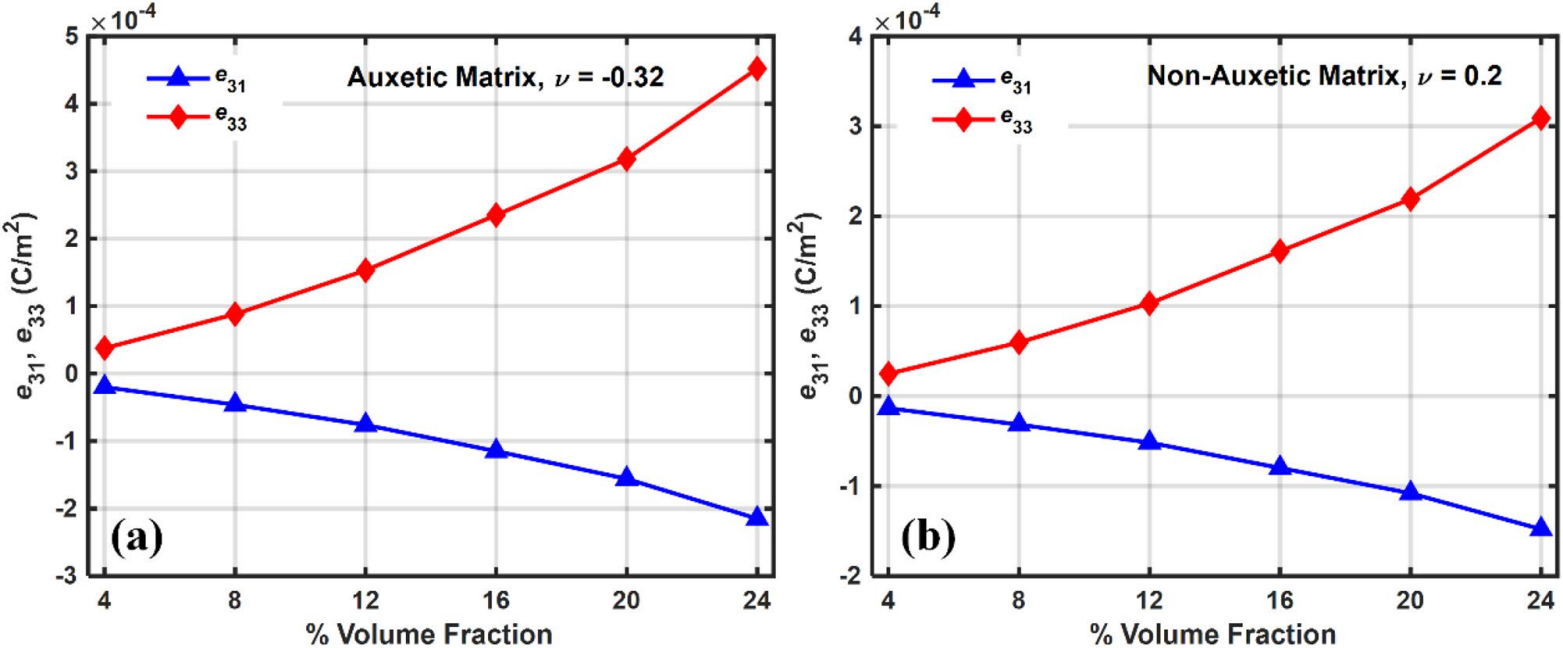
Plot of effective piezoelectric coefficients vs. volume fraction for (**a**) Auxetic material (**b**) Non-Auxetic material.

The effective stiffness parameters of the piezocomposite with auxetic and non-auxetic matrices at different volume fractions are shown in Fig. 6. Mixture rule and higher stiffness of inclusions explain the increase in stiffness of piezocomposite with volume fraction. A similar pattern can be observed in the case of effective piezoelectric properties ${e}_{31}^{eff}$ and ${e}_{33}^{eff}$, as shown in Fig. 7 and is also explained by the rule of mixtures.

The Poisson's ratio was found to have an insignificant effect on the relative permittivity (${\varepsilon }_{s}$ measured at constant strain) of the piezocomposite material; this is to be expected since the stress and stiffness terms are not involved in the determination of the effective permittivity.

The relative permittivity is calculated using the boundary conditions 10 and 11 given in Table 1. Neither the stress terms ${\overline{T} }_{ij}$ and nor the stiffness coefficients ${C}_{ij}^{eff}$ are involved in the calculation of the effective relative permittivity. This means that Poisson's ratio affects only the stiffness parameters and not the electric field $\left({\overline{E} }_{1}\right)$ and the electric displacement $\left({\overline{D} }_{1}\right)$^98^, which explains why Poisson’s ratio has an insignificant effect on the relative permittivity of the piezocomposite. Sensing and energy harvesting was carried out both in transverse mode (${d}_{31}$ mode) and longitudinal mode (${d}_{33}$ mode).

**Figure 8:**
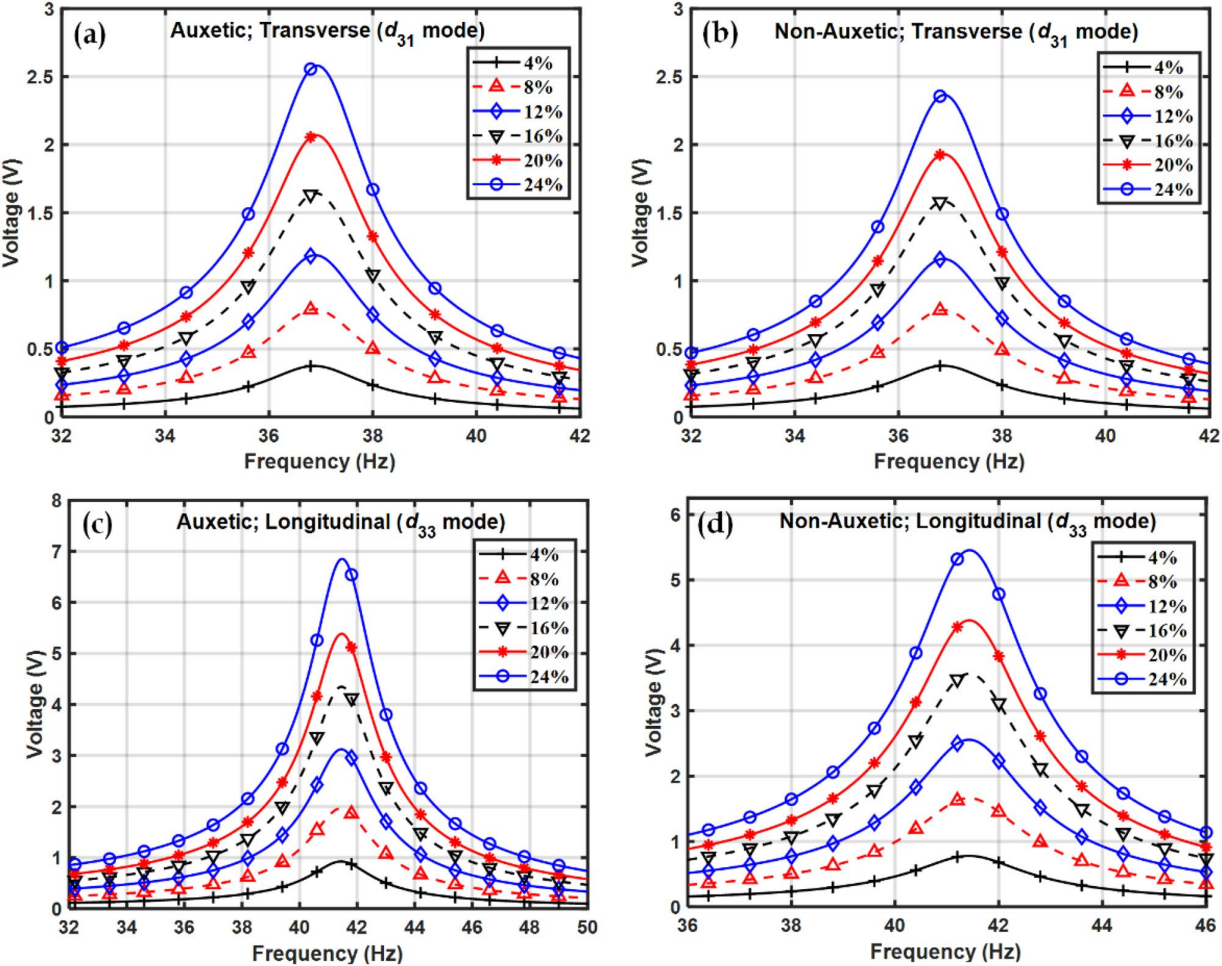
Voltage obtained from piezocomposite in the frequency domain at different volume fractions of BCZT in transverse $\left({d}_{31}\right)$ mode for (**a**) Auxetic, (**b**) Non-Auxetic piezocomposite and in longitudinal $\left({d}_{33}\right)$ mode of (**c**) Auxetic and (**d**) Non-Auxetic piezocomposite.

**Figure 9:**
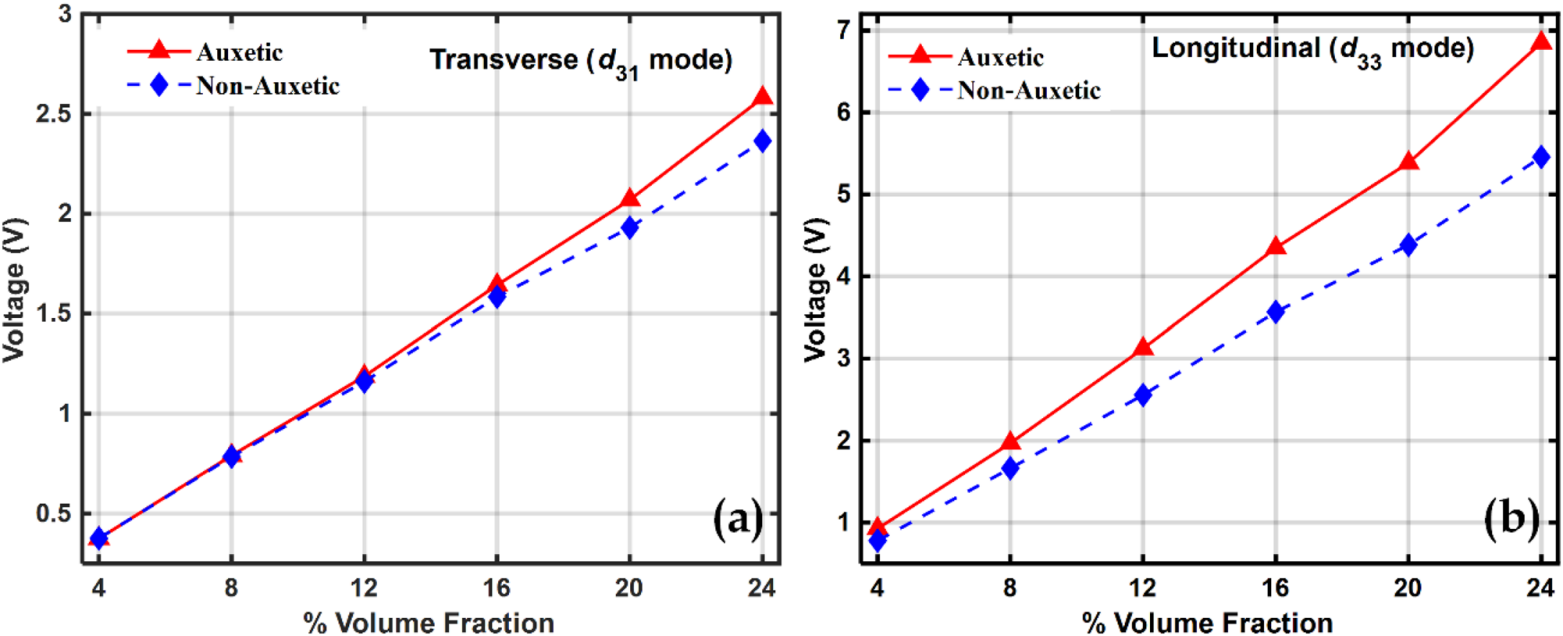
Maximum voltage at different volume fractions of BCZT in (**a**) transverse $\left({d}_{31}\right)$ mode and (**b**) longitudinal $\left({d}_{33}\right)$ mode.

The cantilever beam-based energy harvester operating in the above two modes was subjected to a base vibration and the responses were collected in the frequency domain. Figure 8 shows the frequency domain plot of the open-circuit voltage, namely the sensing capability, obtained from piezocomposites at different volume fractions of BCZT in the frequency domain. Figure 9 shows the maximum values of the open circuit voltage, or sensing voltage, at different volume fractions.

The open-circuit voltage for both the piezocomposites are shown operating both at ${d}_{31}$ and ${d}_{33}$ mode. The sensing voltage was found to increase with an increase in the volume fraction of the piezoelectric material and this can be explained by the fact that as the volume fraction increases, the piezoelectric coefficients also increase with the volume fraction, as shown in Fig. 7. Compared to non-auxetic, a significant improvement in sensing voltage was observed in auxetic piezocomposite. In the transverse and longitudinal configuration, the auxetic piezocomposite reported 2.4 and 1.4 times more sensing voltage than the non-auxetic one.

At 24% volume fraction it can also be observed that voltage in ${d}_{33}$ mode is much higher than the voltage in ${d}_{31}$ mode. The electrode arrangement in the transverse and longitudinal configuration are Top Bottom Electrode (TBE) and interdigitated electrode (IDE) arrangement respectively. It has been observed that the capacitance in the longitudinal ${d}_{33}$ mode is always lower than that in transverse ${d}_{31}$ mode and this could be the reason why the open-circuit voltage is higher in longitudinal $\left({d}_{33}\right)$ mode compared to that in transverse $\left({d}_{31}\right)$ mode^99^; since the relationship between charge, voltage and capacitance is given by *Q* = *CV*. The IDE arrangement of the electrodes, as opposed to the TBE arrangement, could be responsible for the lower capacitance observed in the longitudinal ${d}_{33}$ mode. It's possible that the electric field lines are curved in the longitudinal ${d}_{33}$ mode because of the side-by-side arrangement, whereas in the transverse ${d}_{31}$ mode the field lines are straight and parallel. This could be the reason why the capacitance is lower in the longitudinal ${d}_{33}$ mode in comparison to the transverse ${d}_{31}$ mode^100,101^.

The energy harvesting performance is studied by connecting the harvester to an external load, in the form of a resistance ${R}_{L}$. The maximum power is harvested at the optimum resistance of the load resistance, which depends upon the natural frequency $\omega$ of the structure and the capacitance $C$ between the electrodes, given by Eq. (46).

46 $$R_{opt} = \frac{1}{\omega C}$$

**Figure 10:**
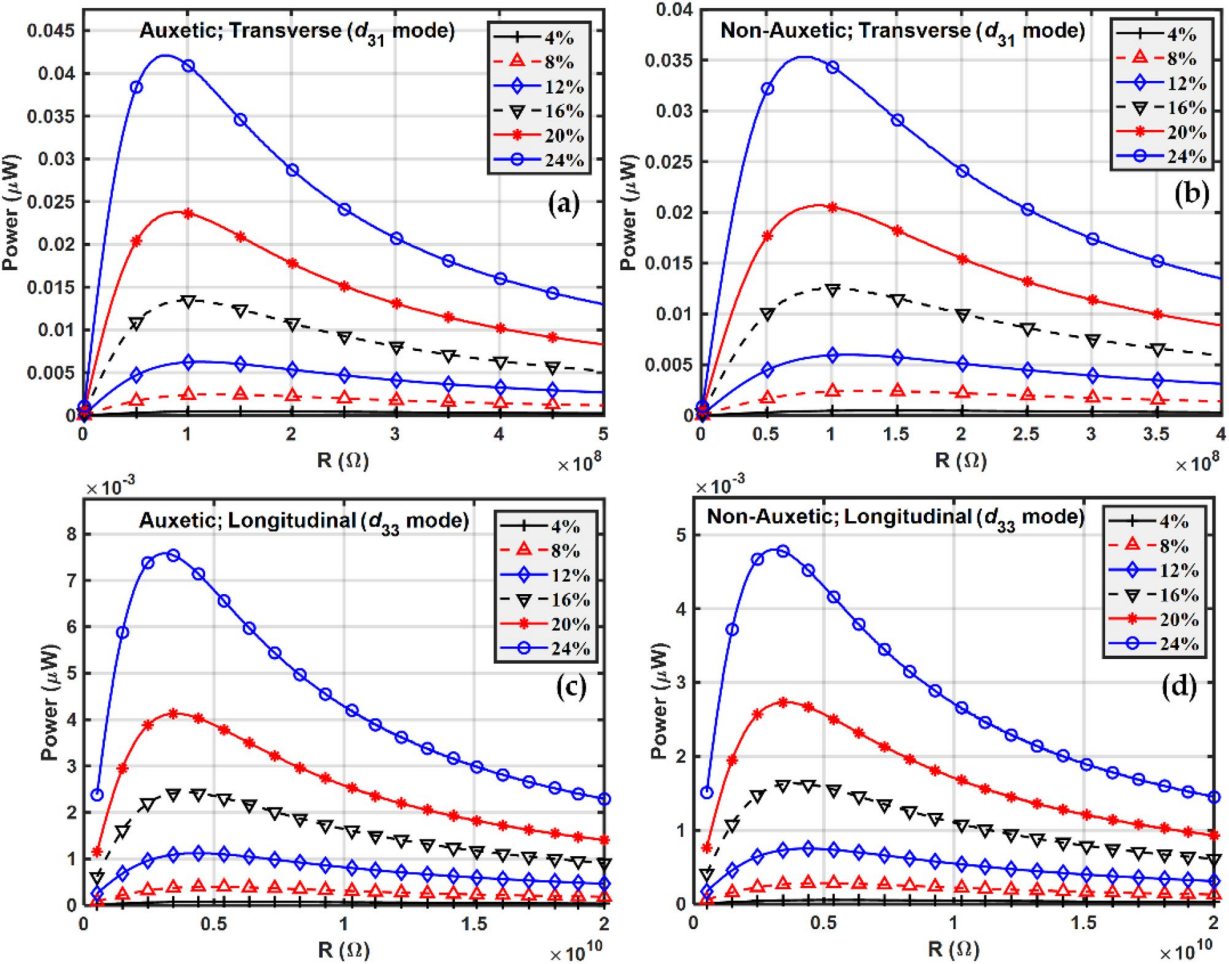
Power vs Resistance at different volume fractions of BCZT in transverse ${d}_{31}$ mode of (**a**) Auxetic (**b**) Non-Auxetic; and in longitudinal $\left({d}_{33}\right)$ mode (**c**) Auxetic (**d**) Non-Auxetic.

A power vs. resistance graph is used to estimate the optimum resistance ${R}_{opt}$ at which maximum power is obtained. Fig. 10 shows the plot of the power vs resistance for both auxetic and non-auxetic matrix-based piezocomposites operating in both the modes, transverse and longitudinal. The harvester and the external load form a $RC$ circuit. Since the capacitance of the piezoelectric material does not change by varying the external load resistance it is possible to determine the optimum resistance at which power attains its maximum value which is evident in the plots shown in Fig. 10. The optimum resistance generally depends upon the natural frequency of vibration $\omega$ of the structure. It can be observed that as the volume fraction of inclusions increases, the harvested power also increases. This is obvious and can be explained by the mixture rule since there are more piezoelectric inclusions present in the composite. It can also be observed that as the volume fraction of inclusion changes, the optimum resistance also changes. This is because with a change in the volume fraction of inclusions both the stiffness and capacitance of the piezocomposite will change; for example, the increase in permittivity and therefore capacitance with increasing inclusion fraction will lead to a reduction in optimum resistance from Eq. (46). The change in stiffness will also affect the natural frequency of vibration of the structure. These factors lead to the observation that as the volume fraction of inclusion changes, the optimum resistance at which maximum power is obtained also changes (as given in Eq. (46)).

**Figure 11:**
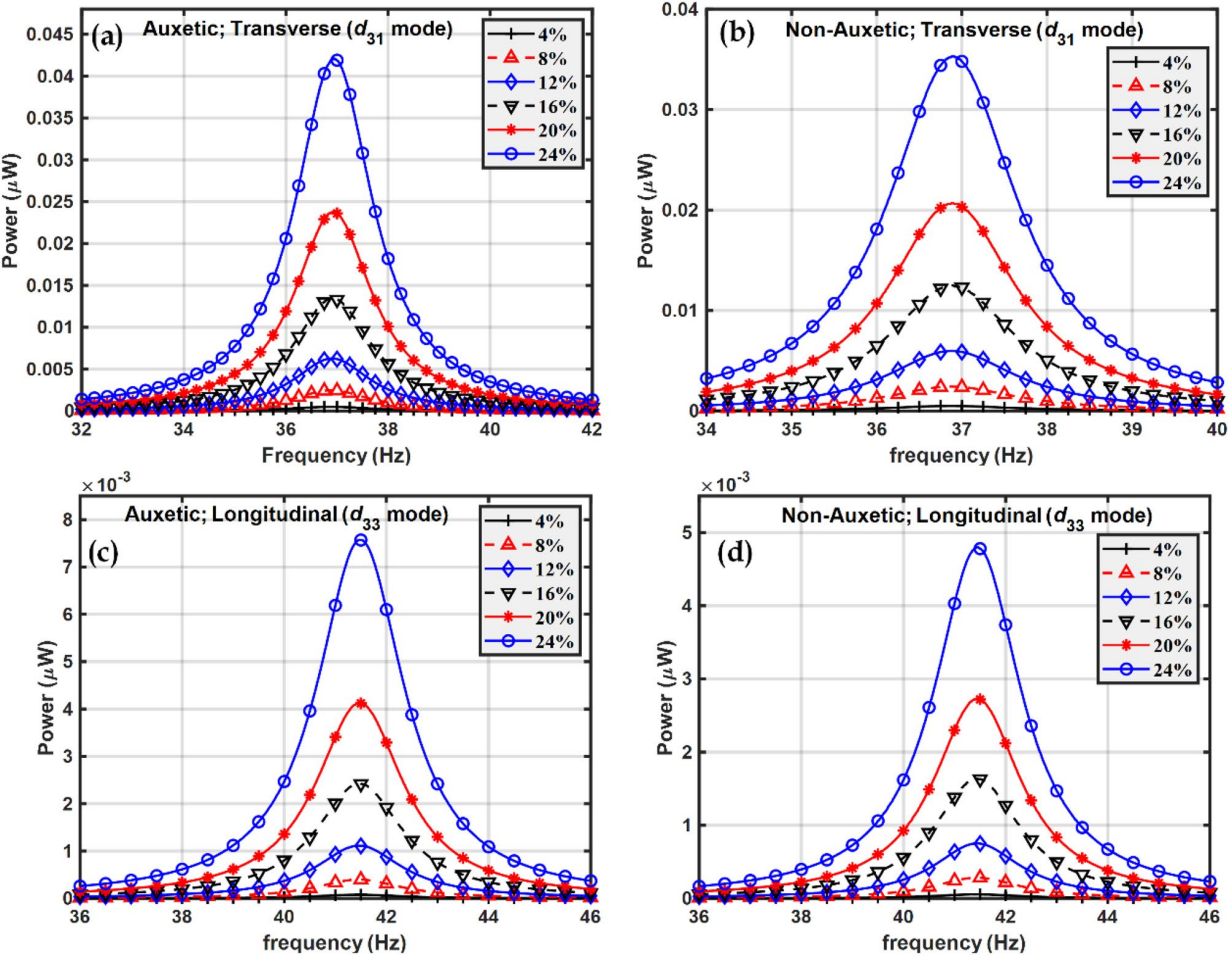
Power in the frequency domain at different volume fractions of BCZT in transverse $\left({d}_{31}\right)$ mode for (**a**) Auxetic and (**b**) Non-auxetic piezocomposite; and in longitudinal $\left({d}_{33}\right)$ mode for (**c**) auxetic and (**d**) non-auxetic piezocomposite.

The optimum resistance ${R}_{opt}$ is obtained from Fig. 10 for the different cases shown and Fig. 11 shows how maximum power varies with frequency at the optimum resistance.

The natural frequency of vibration was not found to vary significantly in the frequency domain plots in Figs. 8 and 11. A possible explanation is that the elastic properties of the host steel beam structure dominate and piezoelectric properties have little effect on the natural frequency of vibration of the structure.

**Figure 12:**
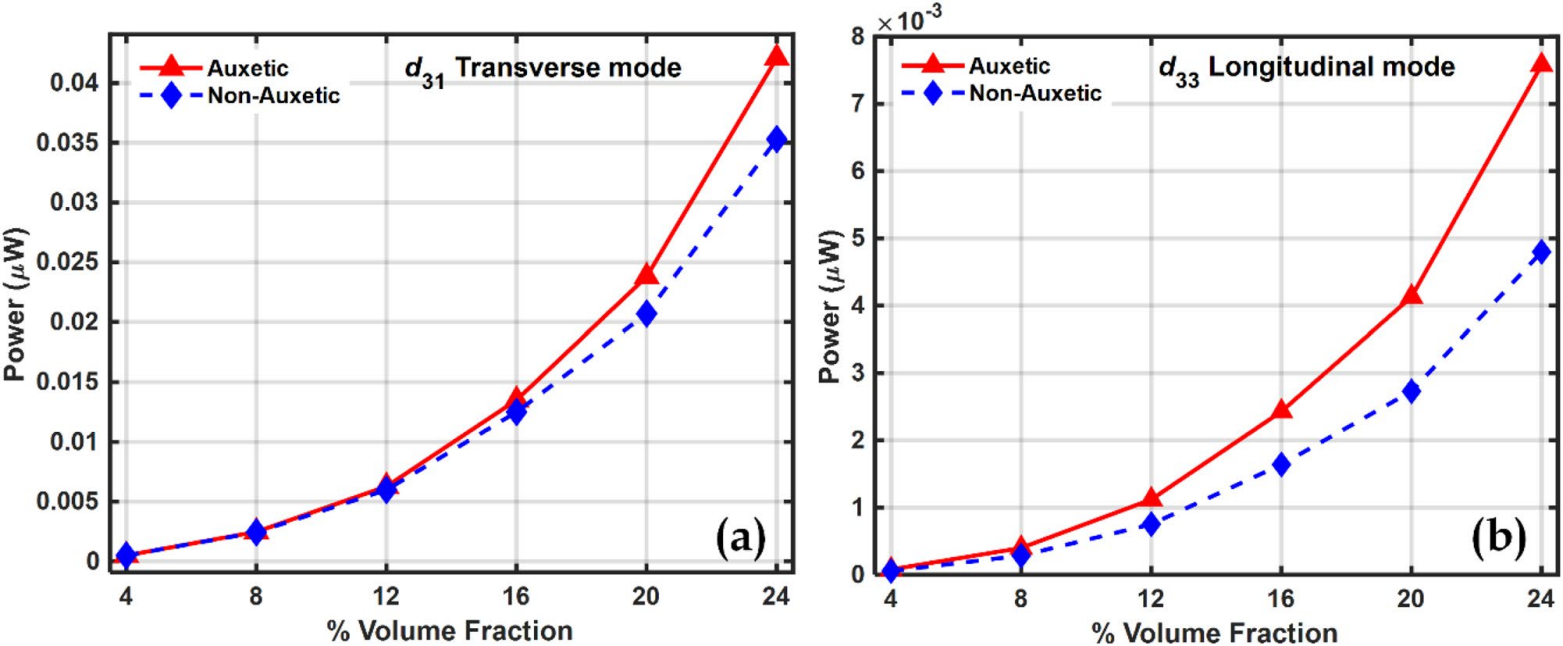
Maximum power vs volume fraction of BCZT auxetic and non-auxetic piezocomposite in (**a**) Transverse mode (**b**) Longitudinal mode.

**Figure 13:**
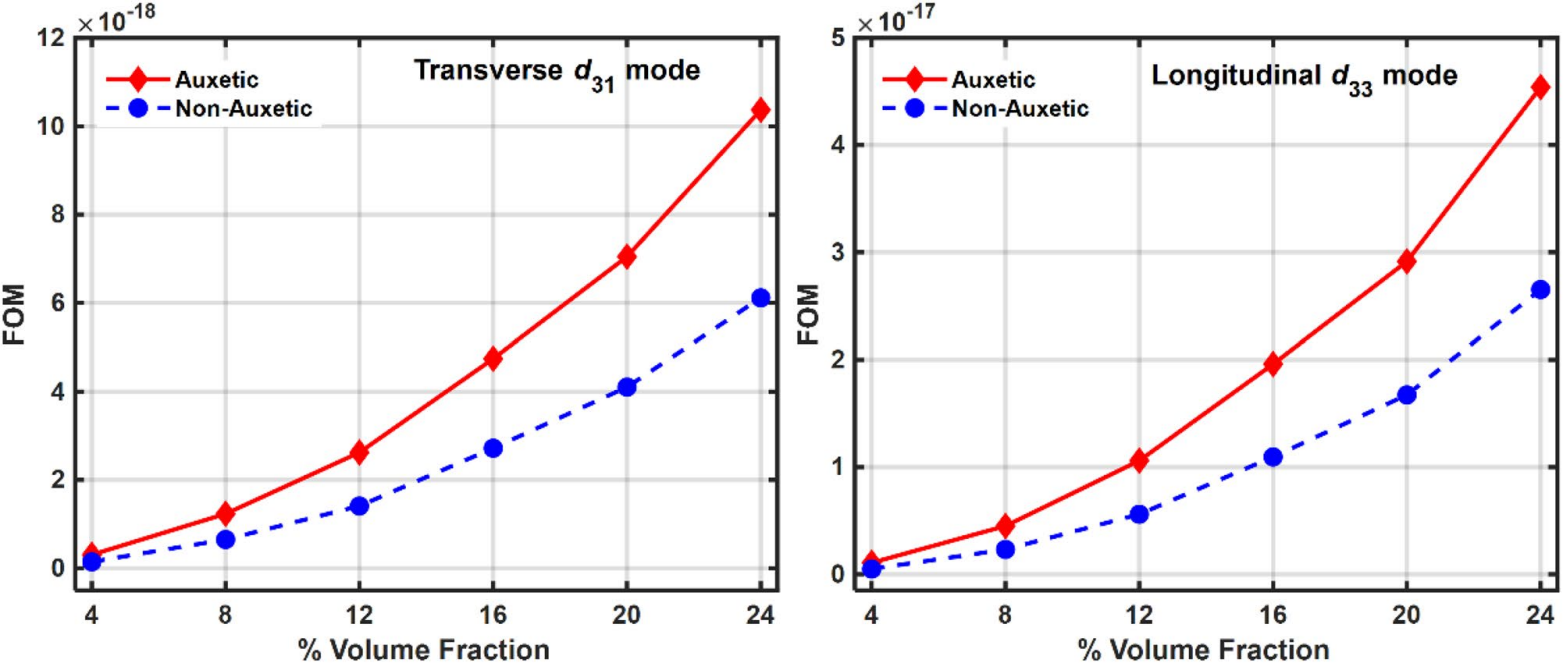
Variation of FOM with volume fraction for both Auxetic and Non-Auxetic materials in (**a**) Transverse $\left({d}_{31}\right)$ mode and (**b**) Longitudinal $\left({d}_{33}\right)$ mode.

As a result, the natural frequency of vibration remains almost the same in both the piezocomposites, auxetic and non-auxetic. From Figs. 10, 11, and 12 it can be observed that as the volume fraction of inclusions increases, the maximum power also increases and this can be explained by the rule of mixtures and increased piezoelectric coefficients. Auxetic materials showed a marked increase in harvested power, compared to their non-auxetic counterpart in both the modes of operation, transverse $\left({d}_{31}\right)$ and longitudinal $\left({d}_{33}\right)$. The auxetic piezocomposite showed an increase in power by approximately $19\%$ in the transverse mode and approximately $58\%$ in the longitudinal mode at a volume fraction of 24%. The figure of merits (FOM) is calculated to validate the harvested power, where the FOM of the energy harvester is given by Eq. (47)^102^

47 $$FOM = \frac{{d^{2} \times E}}{\varepsilon }$$

where $d$ is the piezoelectric coefficient, $E$ is Young’s modulus and $\varepsilon$ is the dielectric permittivity. Fig. 13 shows the figures of merit calculated at different volume fractions. Comparative analysis of Figs. 12 and 13 explain the pattern of the rise of maximum power with volume fraction.

At higher volume fractions the effective properties increase. However, their relative rate of increase decides the nature of the FOM graph. The faster rate of increase in the numerator compared to the denominator in Eq. (47) explains why FOM increases as the volume fraction rises. Correspondingly, the maximum power increases accordingly.

**Figure 14:**
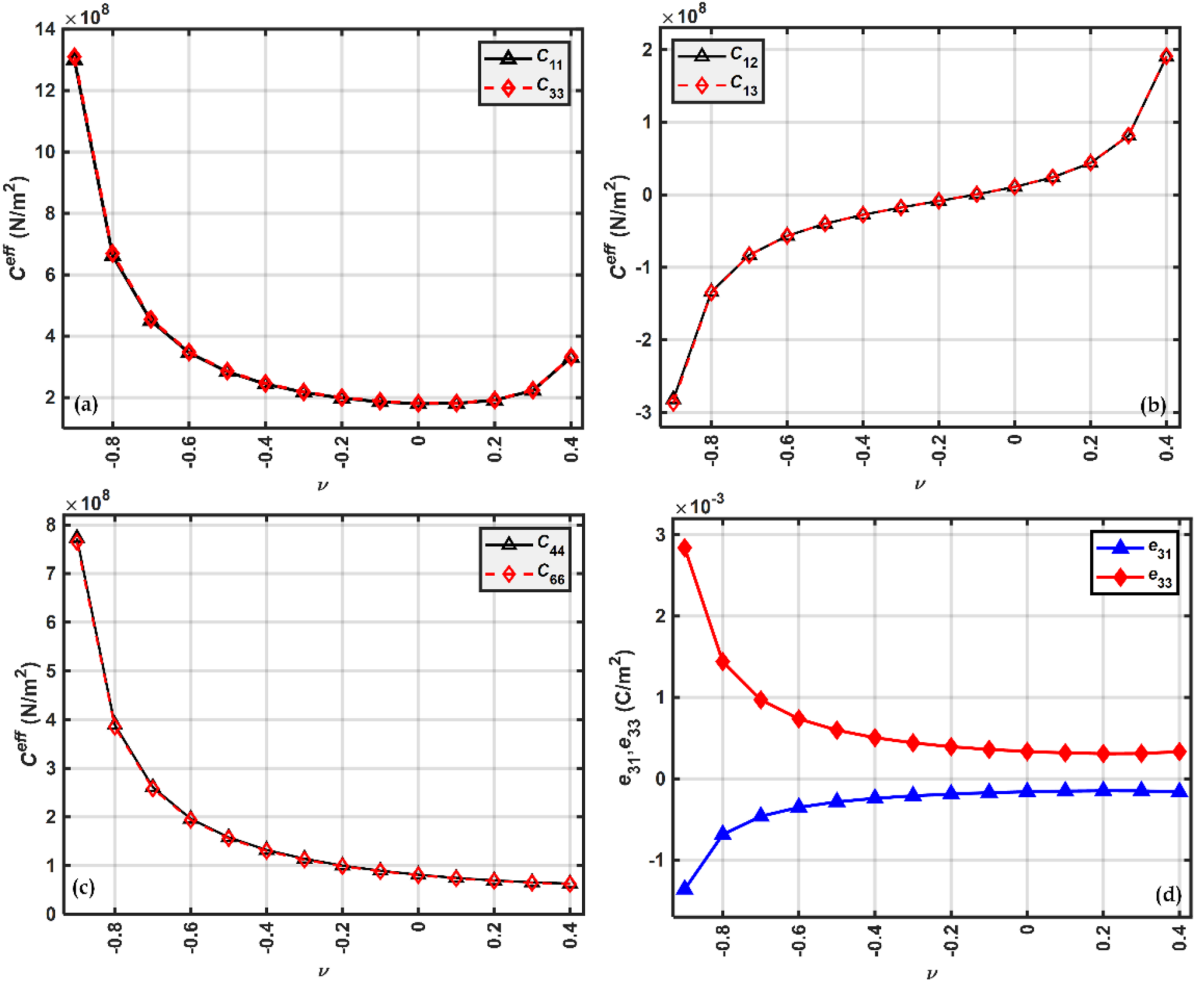
Effective stiffness and piezoelectric properties variation with Poisson's ratios (**a**) ${C}_{11},{C}_{22}$ (**b**) ${C}_{12}, {C}_{13}$ (**c**) ${C}_{44}, {C}_{66}$ and (**d**) ${e}_{31}, {e}_{33}$.

**Figure 15:**
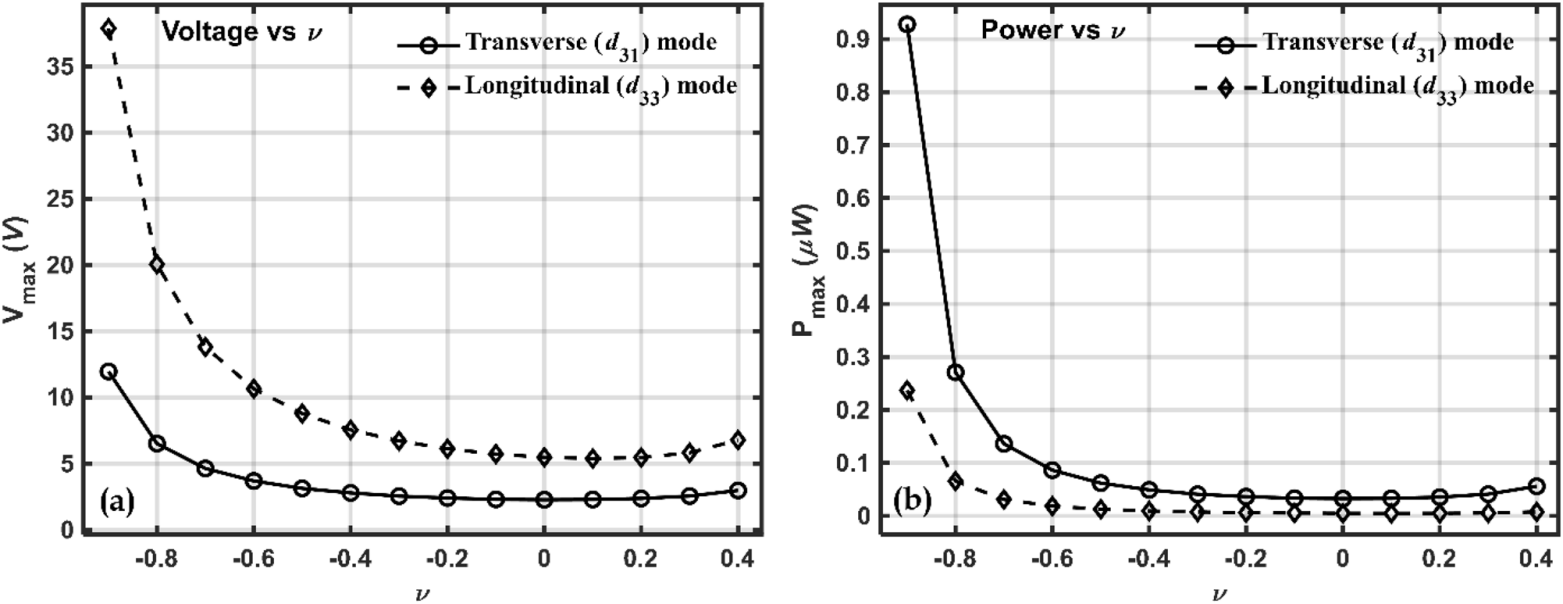
Plot of (**a**) maximum voltage and (**b**) maximum power w.r.t Poisson’s ratio in Transverse $\left({d}_{31}\right)$ and Longitudinal $\left({d}_{33}\right)$ mode.

To explore further the effect of Poisson's ratio on the energy, the Poisson's ratio is varied theoretically between −0.9 to 0.4. Negative Poisson's ratio, as large as $\nu =-0.9,$ has been mentioned^103,104^. The effective elastic/stiffness and piezoelectric properties at $24\%$ volume fraction is calculated as shown in Fig. 14a–d. Fig. 15a shows the variation of sensing voltage with Poisson’s ratio in both the modes, ${d}_{31}$ and ${d}_{33}$ modes. Larger voltage in transverse mode could be because this configuration has a larger capacitance than the longitudinal mode^99^.

In ${d}_{31}$ configuration, the voltage increased by approximately 4 times and in the longitudinal mode, it is increased by approximately 5.6 times. Assuming that the external loads will remain constant when the beam is subjected to base vibrations, the deflection will depend upon the stiffness/elastic properties shown in Fig. 14a–c. The sensing voltage pattern is shown in Fig. 15a and is due to the cumulative effect of all these properties.

Fig. 15b shows the variation of power harvested at optimum resistance with Poisson's ratio in the two modes of operation, transverse and longitudinal. In the transverse mode (*d*_31_) the auxetic piezocomposite at $\nu =-0.9$ generated approximately 16% more power than the non-auxetic piezocomposite at $\nu =0.4$. The corresponding increase in the longitudinal mode (*d*_33_) is ~ 32%. Larger capacitance in transverse (*d*_31_) mode leads to accumulation of more charges and hence more current in *d*_31_ configuration compared to that in *d*_33_ configuration. This is due to the accumulation of more charges and current in the *d*_31_ configuration than in the *d*_33_ configuration.

**Figure 16:**
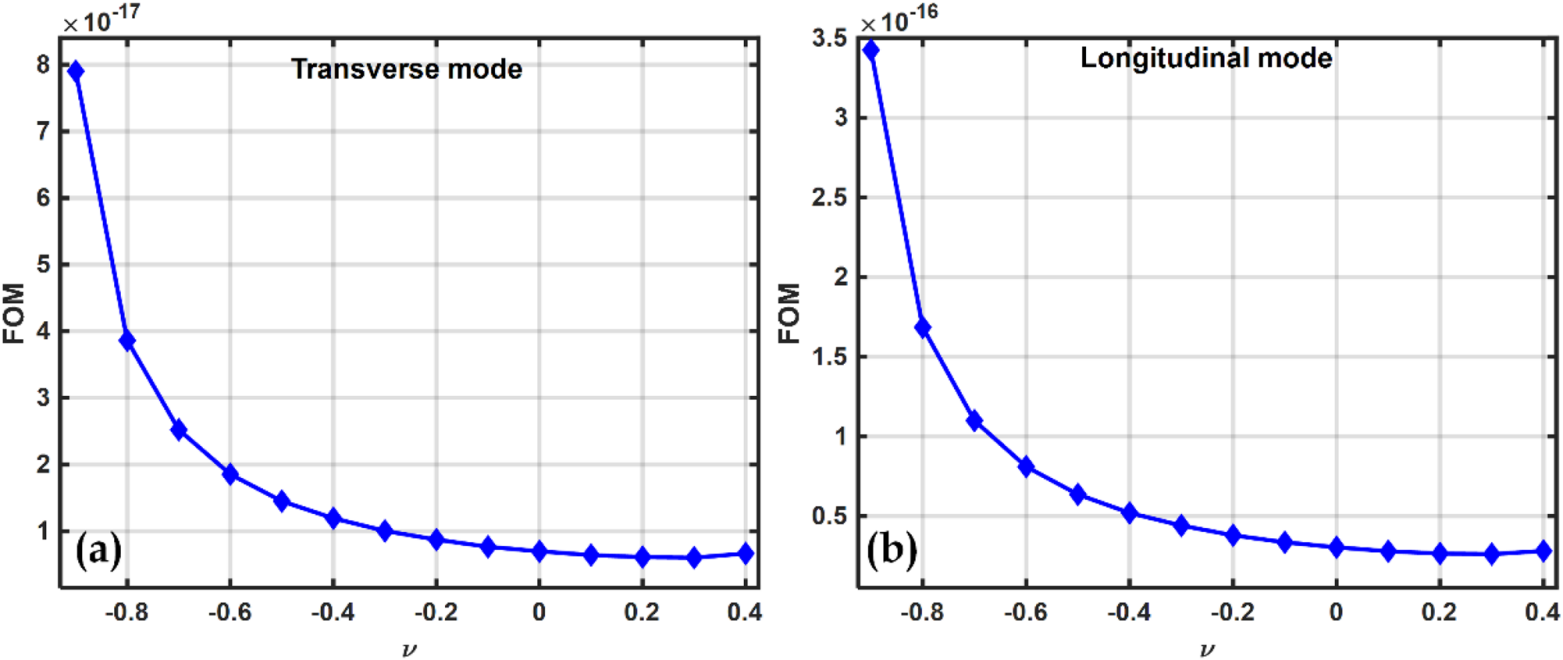
FOM vs $\nu$ (**a**) Transverse $\left({d}_{31}\right)$ mode and (**b**) Longitudinal $\left({d}_{33}\right)$ mode.

Since power depends upon the square of the current term i.e., $P={i}^{2}{R}_{L}$, therefore, ${d}_{31}$ configuration gives higher power than ${d}_{33}$. This can also be explained by the relative variation of material properties with volume fractions as can be observed from the similarity in FOM (Eq. (47)) and harvested power plots with Poisson's ratio as shown in Figs. 15b and 16. It is possible that the pattern of harvested power and the FOM pattern with volume fraction will always match, but the magnitude of the harvested power may change depending on the dimensions of the piezoelectric patch and the electrode separation. This is due to the fact that, in contrast to FOM calculations, which are entirely dependent on the material properties and are independent of the geometries of the vibrating device and the piezoelectric patch, the harvested power in both the ${d}_{31}$ and ${d}_{33}$ modes not only depends on the material properties but is also dependent on the dimensions, effective areas, and electrode spacings of the piezoelectric patch that is attached to the host vibrating structure as well as on the host vibrating structure itself^99,102,105,106^.

**Figure 17:**
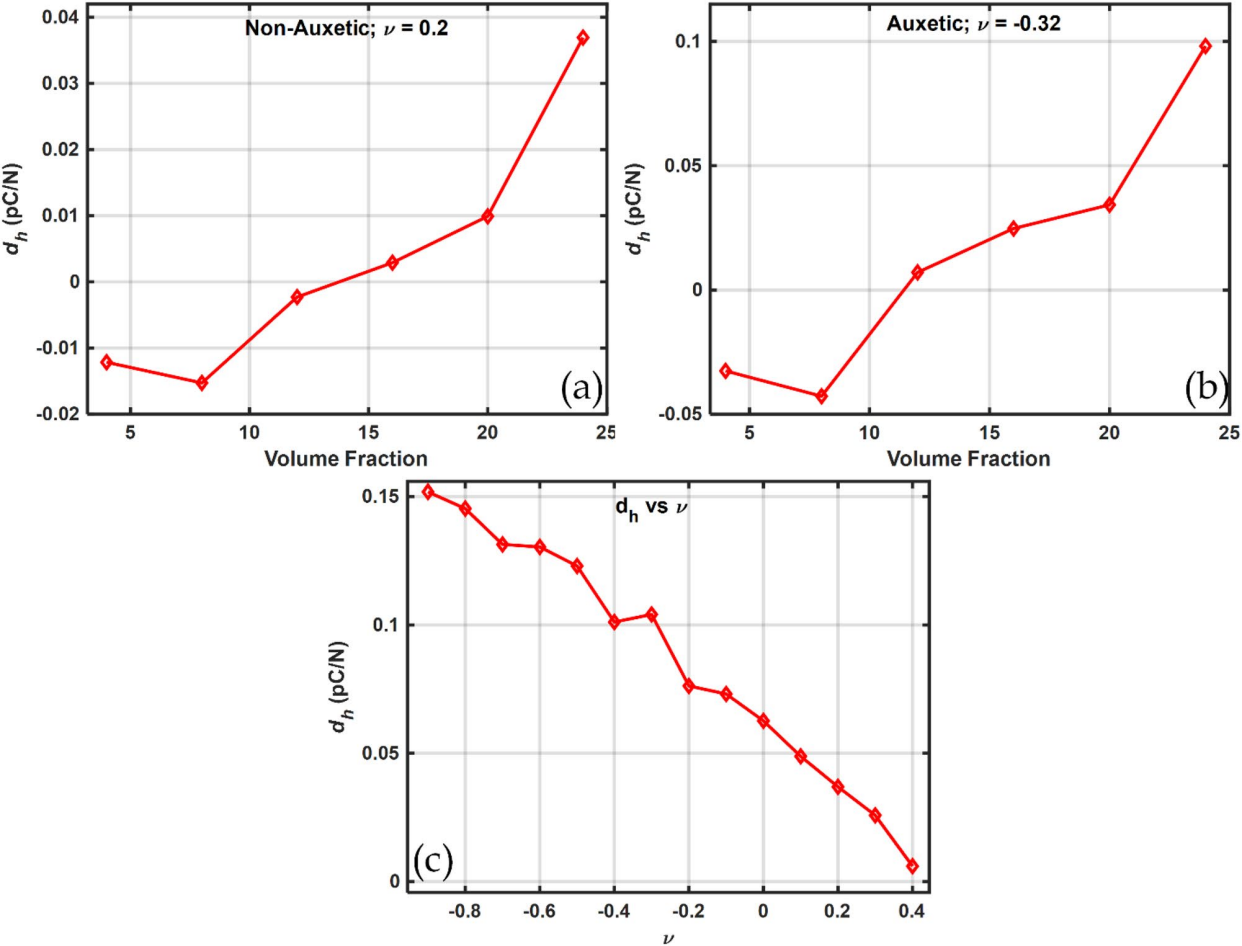
Hydrostatic charge coefficient ${d}_{h}$ calculated for (**a**) Non-Auxetic, (**b**) Auxetic piezocomposite and, (**c**) in the Poisson’s ratio range of −0.9 to 0.4.

Figure 17 shows the hydrostatic charge coefficient at different volume fractions (Fig. 17a,b) and at different Poisson's ratios (Fig. 17c) at a volume fraction of $24\%$. The hydrostatic charge coefficient is calculated using the relation (48).

48 $$d_{h} = 2d_{31} + d_{33}$$

In accordance with the rule of mixture, the magnitude of the effective properties continues to increase in tandem with an increase in the volume fraction of piezoelectric inclusions contained within the polyethylene matrix. Composite material properties can be predicted using the rule of mixtures which is a weighted mean of the properties of the individual constituents^107,108^. The weighing factor that is most commonly used to calculate the weighted means are area fraction and volume fraction.

As the volume fraction of the piezoelectric inclusions increases within the polyethylene matrix, the hydrostatic coefficient decreases first and then increases, as shown in Fig. 17a,b. This is due to the fact that the transverse mode coefficient (*d*_31_) is negative while the longitudinal mode coefficient (*d*_33_) is positive. At 8% volume fraction, the decrease in transverse coefficient (*d*_31_) takes precedence over the increase in longitudinal coefficient (*d*_33_), causing the hydrostatic coefficient *d*_*h*_ to decrease first. Beyond this point, at higher volume fractions of 12% and above, the increase in the longitudinal coefficient (*d*_33_) dominates over the decrease in the transverse coefficient (*d*_31_) resulting in a consistent increase in the hydrostatic coefficient *d*_*h*_ with increasing volume fraction of the piezoelectric inclusions within the polyethylene matrix.

However, the use of auxetic matrix significantly improved the hydrostatic charge coefficient ${d}_{h}$. This is further confirmed by Fig. 17c where the maximum value of the hydrostatic coefficient is observed at the negative end of the Poisson’s ratio spectrum at $\nu =-0.9$. The improved and superior performance of the auxetic piezocomposite over the non-auxetic one could be due to more effective strain transfer to the piezoelectric inclusions in the case of auxetic piezocomposite than in the non-auxetic. This is due to the superior mechanical coupling of the strain applied to the rigid BCZT piezoelectric inclusions embedded in the soft polyethylene matrix^52,109^.

Table 2 lists the previous works related to auxetic composites and piezocomposites and corresponding improvements achieved. Achievement in the present work in comparison to the previous is also presented.

**Table 2:** Previous work done on Auxetic materials.

Sl no	Literature	Auxetic type (structure/material)	-${\nu }_{min}$	Study Type	Improvements/achievements
1	Lakes^104^	NPR Auxetic foam	−1.0	Experimental	Improved resilience & toughness
2	Miki^110^	Laminated Fibrous Composite	−0.37	Analytical/experimental	Laminate orientation & NPR Correlation
3	Milton^111^	Isotropic NPR, Structure based/Two phase composites	−1	Analytical/mathematical Model	Isotropic materials with $\nu =-1$ achieved
4	Nkansah ^112^	Fibre reinforced composite with NPR	−0.9	Numerical study (FEM)	Enhanced transverse modulus of the composite
5	Alderson ^113^	Auxetic Composites	−0.16	Experimental Studies	Enhanced mechanical properties
6	Alderson ^51,53^	Auxetic Polyethylene Matrix	−0.32	Experimental Studies	Improved flexural properties, large indentation resistance
7	Subramani^114^	Auxetic structure composites	−5.20	Analytical model/experimental	High strength/High energy absorption capacity
8	Krishnaswamy et al.^52^	Inherently and designed structures auxetic piezocomposite	−0.32	Numerical/experimental Study	Improved piezoelectric Properties
9	Karmakar et al.^97^	KNNNS-BNZH auxetic polyethylene based piezocomposite	−0.9	Numerical/ FEM	Improved sensing and energy harvesting performance
10	Present Work	BCT-BZT auxetic polyethylene based piezocomposite	−0.9	Numerical/ FEM	

## Assumptions and Limitations

Finite element analysis-based comparative study was conducted to study auxetic and non-auxetic piezocomposite for sensing and energy harvesting applications.

Finite element method is a popular approximate solution method that provides solutions to many practical engineering problems. However, this study is based on certain assumptions which limit the scope of the study. Those assumptions and limitations should therefore be kept in mind while implementing this kind of model. A unit cell model, where the RVE is assumed to be a unit cube, has been used to predict the effective properties of the piezocomposite. The above model assumes that the properties of the entire material represent the average property of the unit cell as a whole. In addition, this model assumes that the effective properties depend on the volume fraction of the inclusion and not on the particle size of the inclusions. Therefore, this model is to be used in those cases where there is a uniform and homogeneous distribution of inclusions within the matrix volume. In addition, care should be taken that the present model only considers the volume fraction of the inclusion and not their size within the RVE. The ambient base vibration to which the beam is subjected is assumed to be very small so that the body loads acting on the beam are assumed to be in the reign of linear piezoelectricity, and not non-linear effects associated with domain motion. The model will not be able to predict other important phenomena and parameters such as electrical breakdown strength, fatigue life, and life cycle of the cantilever beam-based energy harvester.

## Conclusion

Finite element analysis was carried out on $0-3$ polyethylene and BCZT based piezocomposite materials to determine the effective properties of the piezocomposite using a homogenization technique. An RVE having unit dimensions, also called a unit cell, is defined, and periodic boundary condition is applied to calculate the effective properties at six different volume fractions (4%, 8%,…, 24%). The harvester performance is evaluated in terms of sensing voltage and harvested power using the calculated values of the effective properties. Improved performance was observed at higher volume fractions and can be explained by the rule of mixtures. An overall performance improvement was observed in auxetic piezocomposite compared to non-auxetic. This is because the strain is transferred to the inclusions in a much better way in the case of the auxetic matrix compared to non-auxetic matrix due to improved mechanical coupling. At a 24% volume fraction, the auxetic piezocomposite $\left(\nu =-0.32\right)$ generated 8% and 25.5% more sensing voltage in transverse and longitudinal modes respectively compared to its non-auxetic counterpart $\left(\nu =0.2\right)$. A corresponding increase in harvested power is approximately 20% and 58% in transverse and longitudinal mode respectively.

Sensing voltage and power were calculated at 24% volume fraction for a range of Poisson's ratio between −0.9 to 0.4. Sensing voltage and the harvested power increased significantly at the negative extreme of the Poisson’s ratio spectrum when Poisson’s ratio is −0.9. Compared to non-auxetic, the auxetic piezocomposite generated 4 and 15.5 times more sensing voltage and power in ${d}_{31}$ configuration. Corresponding increment in the ${d}_{33}$ configuration was about 5.6 and 34 times respectively. The above studies conclude that piezocomposites made of negative Poisson's ratio metamaterial can significantly improve the voltage and power output of the harvester.

## Figures and Tables

**Figure 18:**
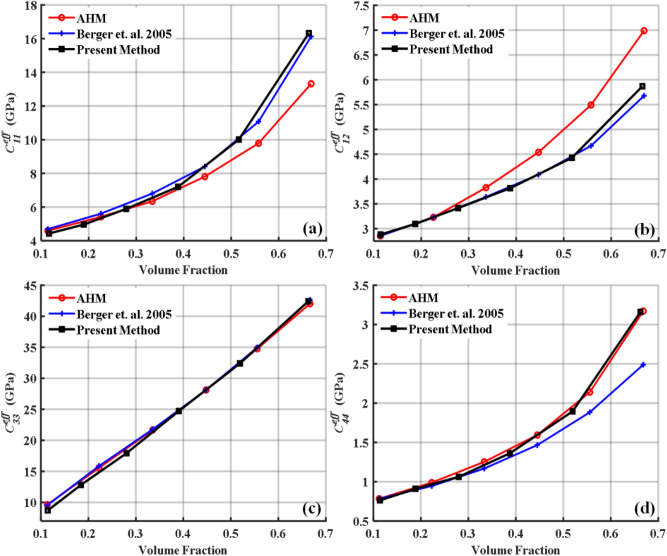
Validation of present model by comparing effective stiffness properties.

**Figure 19:**
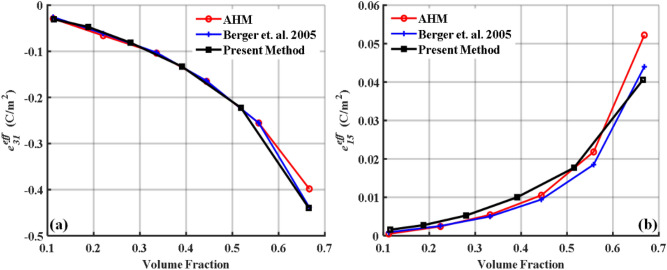
Validation of the present model by comparing the effective piezoelectric properties.

**Figure 20:**
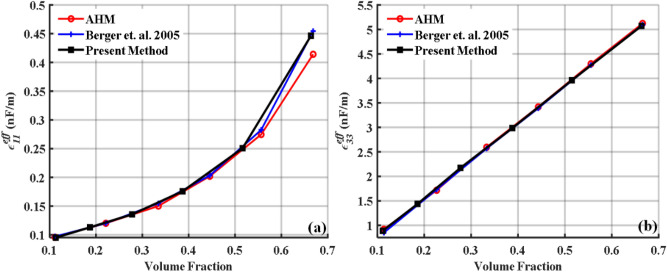
Validation of present model by comparing effective relative permittivity.

**Figure 21:**
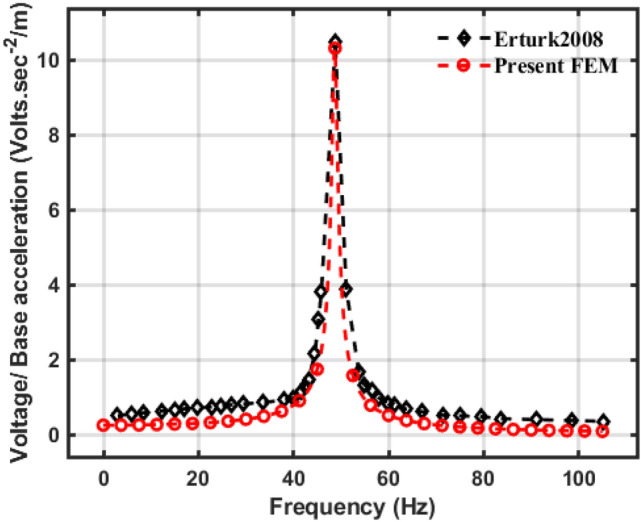
Validation of energy harvester model by comparing the voltage per unit base acceleration.

## Data Availability

All data generated or analysed during this study are included in this published article [and its supplementary information files].

## Appendix

The finite element model used in the present work is validated by comparing the effective properties of piezoelectric fibers (PZT-5) embedded in a polymer matrix by Finite Element Method (FEM) and Asymptotic Homogenization Method (AHM) by Berger et al.^72^ The results obtained by the present FEM method were found to be in good agreement with FEM and AHM method reported in Berger et al.^72^.

Figures 18, 19, and 20 compare the effective properties calculated by the present FEM method and by Berger et al.^72^. The present FEM model for the piezoelectric energy harvester is validated by comparing the voltage generated per unit base acceleration for a PZT-5A piezoelectric material attached to a host structure, as given in Erturk and Inman^115^. The parameters of the piezoelectric material and the host beam, taken from Erturk and Inman^115^ are given in Table

**Table 3:** Material properties of host beam and piezoelectric patch for validating the energy harvester model.

Piezoelectric material properties (PZT-5A)	Host material (metal beam)
${C}_{11}^{E}={C}_{22}^{E}=120.3 \mathrm{GPa}$	Young’s Modulus $E=100 \mathrm{GPa}$
${C}_{33}^{E}=110.9 \mathrm{GPa}$	Mass Density = $\rho =7165 \mathrm{kg}/{\mathrm{m}}^{3}$
$${C}_{12}^{E}=75.2 \mathrm{GPa}$$	
${C}_{13}^{E}={C}_{23}^{E}=75.1 \mathrm{GPa}$	Poisson’s ratio = $\nu =0.3$
$${C}_{66}^{E}=22.7 GPa$$	
${e}_{31}^{E}={e}_{32}^{E}=-5.2 (\mathrm{C}/{\mathrm{m}}^{2})$	Rayleigh coefficients
${e}_{33}^{E}=15.9 \left(\mathrm{C}/{\mathrm{m}}^{2}\right)$	$\alpha =4.886 \mathrm{rad}/\mathrm{s}$
Mass density = $\rho =7800 \mathrm{kg}/{\mathrm{m}}^{3}$	$\beta =1.2433\times {10}^{-5} (\mathrm{s}/\mathrm{rad})$
Permittivity = ${\epsilon }_{r}=15.94 \left(\mathrm{nF}/\mathrm{m}\right)$	

3. Figure 21 shows a comparison plot of voltage calculated per unit base acceleration by the present FEM method and by Erturk and Inman^115^. It can be observed that voltage calculated per unit base acceleration calculated by Erturk and by present FEM almost matches with each other, hence validating the present FEM model.
